# Evaluation of New Reference Genes in Papaya for Accurate Transcript Normalization under Different Experimental Conditions

**DOI:** 10.1371/journal.pone.0044405

**Published:** 2012-08-31

**Authors:** Xiaoyang Zhu, Xueping Li, Weixin Chen, Jianye Chen, Wangjin Lu, Lei Chen, Danwen Fu

**Affiliations:** State Key Laboratory for Conservation and Utilization of Subtropical Agro-bioresources/Guangdong Key Laboratory for Postharvest Science and Technology, College of Horticulture, South China Agricultural University, Guangzhou, P.R. China; Soonchunhyang University, Republic of Korea

## Abstract

Real-time reverse transcription PCR (RT-qPCR) is a preferred method for rapid and accurate quantification of gene expression studies. Appropriate application of RT-qPCR requires accurate normalization though the use of reference genes. As no single reference gene is universally suitable for all experiments, thus reference gene(s) validation under different experimental conditions is crucial for RT-qPCR analysis. To date, only a few studies on reference genes have been done in other plants but none in papaya. In the present work, we selected 21 candidate reference genes, and evaluated their expression stability in 246 papaya fruit samples using three algorithms, geNorm, NormFinder and RefFinder. The samples consisted of 13 sets collected under different experimental conditions, including various tissues, different storage temperatures, different cultivars, developmental stages, postharvest ripening, modified atmosphere packaging, 1-methylcyclopropene (1-MCP) treatment, hot water treatment, biotic stress and hormone treatment. Our results demonstrated that expression stability varied greatly between reference genes and that different suitable reference gene(s) or combination of reference genes for normalization should be validated according to the experimental conditions. In general, the internal reference genes *EIF* (Eukaryotic initiation factor 4A), *TBP1* (TATA binding protein 1) and *TBP2* (TATA binding protein 2) genes had a good performance under most experimental conditions, whereas the most widely present used reference genes, *ACTIN* (Actin 2), *18S rRNA* (18S ribosomal RNA) and *GAPDH* (Glyceraldehyde-3-phosphate dehydrogenase) were not suitable in many experimental conditions. In addition, two commonly used programs, geNorm and Normfinder, were proved sufficient for the validation. This work provides the first systematic analysis for the selection of superior reference genes for accurate transcript normalization in papaya under different experimental conditions.

## Introduction

Gene expression analysis is an important step to understand the roles of genes in developmental and cellular processes, such as the signaling and metabolic pathways [1]. Real-time reverse transcription PCR (RT-qPCR) has emerged as the most widely used method to quantify changes in gene expression profiles in response to different environmental conditions. It has shown important attributes such as accuracy, precision and relative ease of use due to its speed, sensitivity and specificity [2], [3]. Nevertheless, to accurately quantify gene expression, several experimental variations, such as quality and amount of starting material, presence of inhibitors in different sample materials, primer design, and RNA extraction and retro-transcription efficiencies, should be taken into account [4]. Therefore, selection of an appropriate normalization strategy is of crucial importance for the acquisition of biologically meaningful data. Among several methods proposed so far [4], [5], the use of one or more reference genes is currently the preferred method of normalization [6]. An ideal reference gene should be expressed at a constant level across various conditions and its expression is assumed to be unaffected by experimental parameters [7], [8]. Moreover, the reference gene and the target gene should have similar ranges of expression in the samples to be analyzed [9]. Genes involved in basic metabolism and maintenance of the cell, e.g. β-actin, glyceraldehyde-3-phosphatede hydrogenase (*GAPDH*), ribosomal subunits and ubiquitin are commonly used as reference genes [10], [11], [12]. However, several reports have demonstrated that there are no universally applicable reference genes with an invariant expression, and that the using of unstable reference gene will lead to inappropriate biological data interpretation [8], [13], [14]. Thus, there is an urgent need to systematically evaluate the stability of potential reference genes for every particular experimental condition prior to their use in RT-qPCR normalization. Meanwhile, several algorithms, such as geNorm [15], NormFinder [16], BestKeeper [17], qBasePlus [18], and RefFinder [19] have been well developed to validate the most stable reference gene(s) from a panel of potential genes or candidate genes under a given set of experimental conditions [20], [21].

Recently, a growing number of reference gene validation attempts have been reported for plants, such as *Brachypodium distachyon* [22], potato [23], sugarcane [24], rice [25], [26], [27], *Petunia hybrid* [28], soybean [29], tomato [30], [31], wheat [32], barley [33], grape [34], poplar [35], coffee [36], *Arabidopsis thaliana* [13], cucumber [37], chicory [38], *pisumsativum* [39], Swingle Citrumelo [40], *Populus* [41], Peanut [42], lichi [43], tobacco [44], banana [20] and citrus [45]. However, there have been no reports on the suitability of reference genes for RT-qPCR studies of differential expression of genes in papaya.

Papaya (*Carica papaya L.*) is the only species within the genus *Carica* and the most commercially important species within the family *Caricaceae* [46] and it has been widely cultivated in tropical and subtropical lowland regions for its nutritional benefits and medicinal applications. Papaya is also the first perennial transgenic fruit variety for commercial application of the world [47], [48]. However, papaya fruit is subject to some problems such as rapid ripening, susceptible to biotic or abiotic stresses, which could result in a high percentage of product loss [46], [47], [49]. Due to all these matters, papaya has been the focus of many studies at physicochemical, biochemical, and molecular levels [46]. The postharvest biology of papaya fruit has been an important aspect of those studies as well [46], [50], [51], [52]. The understanding of expression patterns of some key genes, especially for the genes associated with ripening and stress responses, will help us to gain insights into the mechanisms involved in these processes, and in turn, to improve fruit quality and storage potential. To date, almost all studies on gene expression in papaya fruit with RT-qPCR have used *Actin* or *18S rRNA* as reference gene [10], [11], [53], [54]. However, the stability of these two genes has not been verified yet and it is not clear whether they are the suitable reference genes in papaya. Therefore, the application of RT-qPCR analyses of gene expression in papaya fruit has been limited by the use of potentially inappropriate reference genes.

For further development of RT-qPCR in papaya, the present study aimed at defining reference genes suited for quantitative analysis of papaya genes under different experimental conditions. Here, we reported a systematic analysis of 21 genes to identify the internal reference gene(s) most suitable for normalization gene expression data obtained with RT-qPCR analysis in papaya. These genes have different roles in the cells, including those involved in cell structure, membrane proteins, transcription, protein translation, protein degradation and metabolic pathways. The data for each gene were obtained from a large set of biological samples representing different experimental conditions, including various tissues, fruit developmental stages, different storage temperatures, different cultivars, postharvest ripening, pathogen stress, 1-MCP treatment, hot water treatment, modified atmosphere packaging (MAP) and hormone treatment. Furthermore, in order to illustrate the usefulness of the newly identified reference genes, expression analysis of one interesting gene related to fruit softening, *CpaEXY1*, was presented. The result provided a superior set of validated reference genes that were suitable for RT-qPCR analysis in papaya fruit under different experimental conditions and clearly indicated that different reference genes should be validated according to the particular experimental conditions.

## Materials and Methods

### Plant Materials and Experimental Conditions

Roots, stems, leaves, flowers, and pre-climacteric papaya fruits at some hint yellow stage (three-line yellow) were freshly harvested from a local commercial plantation nearby Guangzhou, south-eastern China. The vegetative tissue samples, such as root, leaves and stem, were taken from young tissue; flowers were harvested at full bloom. At each sampling time, plant materials except for fruits were immediately frozen in dry ice after harvesting, transported to the laboratory and then stored at −80°C until total RNA was isolated. For samples of different development stages, papaya fruits were sampled at 1, 2, 3, 4 and 5 months after anthesis.

For samples of different cultivars, different developmental stages, postharvest ripening, stresses, 1-methylcyclopropene (1-MCP) treatment, hormone treatment, MAP treatment and hot water treatments, pre-climacteric papaya fruit at the first sign of color break (<10% skin yellow stage) were harvested. Fruits free from visual symptoms of any disease or blemishes were randomly selected for uniformity of weight, shape, and maturity. The selected fruits were firstly cleaned, dipped in a 1% hypochloride solution for 1 min for contraction wounds and then soaked in 0.2% (w/v) Sporgon solution (Bayer, Leverkusen, Germany) for 10 min to eliminate potential microbes. They were then allowed to air-dry at 25°C for 3 h and treated as described below.

For samples of different cultivars and postharvest ripening samples, three widely cultivated and consumed cultivars in south China, ‘*Shuiyou 2′*, ‘*Hongri 1′* and ‘*Hongri 3′*, were chosen. After being harvested and pre-handled, all groups were placed into unsealed plastic bags (0.02 mm thick) and stored at 25°C. Samples were taken differently because of their differential postharvest metabolism. Samples of ‘*Shuiyou 2′* were taken at 0, 2, 4, 6, 8, 10 and 12 days, whereas ‘*Hongri 1′* and ‘*Hongri 3′* were taken at 0, 2, 3, 4, 5 and 6 days after storage.

For different storage temperature samples, four storage temperatures, 7°C, 15°C, 25°C, and 35°C, were set. After harvested and pre-handled, the selected papaya fruits were placed into unsealed plastic bags and transferred to 7°C, 15°C, 25°C and 35°C for preservation, respectively. Samples of 25°C storage were taken at 0, 2, 4, 6, 8, 10 and 12 days, and the samples of 35°C storage were taken at 0, 2, 4, 6, 8 and 10 days after treatment. For storage of 7°C and 15°C, samples were taken at 0, 4, 8, 12, 16, 20 and 24 days after treatment.

For biotic stress samples, the selected papaya fruits were inoculated with 20 ul (4×10^6^ spores ml^−1^) of *Colletotrichum gloeosporioides* Penz. spores in suspension as described by De Capdeville, et al. [52], covered with wet cotton and sealed with bag but open after 24 hours. Fruits were placed into unsealed plastic bags and stored at 25°C. Samples were taken at 0, 2, 4, 6, 8, 10 and 12 days after treatment.

For 1-MCP treatment, papaya fruits were sealed in the closed airtight containers, and 300 µl/L of 1-MCP were injected into the containers through a rubber septum. Fruits were incubated with 1-MCP for 16 h at 25±1°C. The containers were then opened to allow ripening in air in the same temperature conditions and fruits were placed into unsealed plastic bags, as well. Samples were taken at 0, 2, 4, 6, 8, 10 and 12 days after treatment.

For hot-water treatment samples, papaya fruits were dipped into hot water (54°C) for 4 min and were taken out and then allowed to air-dry at 25°C. After that fruits were placed into unsealed plastic bags and stored at 25°C. Samples were taken at 0, 2, 4, 6, 8 and 10 days after treatment.

For samples of modified atmosphere packaging (MAP) treatment, the selected papaya fruits were packed with thick of 0.02 mm thick PE bags, sealed by capper and stored at 25°C. Samples were taken at 0, 3, 6, 9, 12, 15 and 17 days after treatment.

For samples of ethephon treatment, papaya fruits were dipped into ethephon aqueous solution (500 µl/L) for 3 min, and then taken out and allowed to air-dry at 25°C. After that the fruits were placed into unsealed plastic bags and stored at 25°C. Samples were taken at 0, 1, 2, 3, 4 and 5 days after treatment.

All experiments were performed using biological triplicates. The information about all the experimental conditions mentioned above are summarized in Table 1, which composed thirteen sample sets for data analysis (Table 1). For all fruit samples, fruit core was excluded and the peel and flesh were chop up, frozen in liquid nitrogen and stored at −80°C until further use.

**Table 1:** Summary of the experimental conditions and samples used in present study.

Experimentalsample sets	Tissue type	Number oftreatments	Biological replicates	Sampling dates	Total number of samples(treatments × replicates × dates)
Different storage temperatures	Fruit	3	3	6×2# 7×1	57
Different tissues	Root, stem, leaf, flower,peel, pulp	1	3	1	18
Developmental stages	Fruit	1	3	5	15
Postharvest ripening	Fruit	3	3	6×2# 7×1	57
MAP	Fruit	1	3	7	21
Hot water treatment	Fruit	1	3	6	18
1-MCP treatment	Fruit	1	3	7	21
Hormone treatment	Fruit	1	3	7	21
Biotic stress	Fruit	1	3	6	18
Total					246

#indicated that the sample dates including two types: two treatments were 6 and one was 7.

### Total RNA Isolation, Quality Control, and cDNA Synthesis

All the frozen tissues were ground in liquid nitrogen for RNA isolation. Total RNA was extracted using the hot borate method of Wan and Wilkins [55] and then treated with DNAseI digestion using the RNAse-free kit (TaKaRa, Japan) to eliminate the potential DNA contamination. The RNA concentration and purity were evaluated by measuring absorbance at 230, 260 and 280 nm, respectively, using a BioPhotometer plus (Eppendorf, Germany). The integrity of the RNA samples was assessed on 1.2% agarose/formaldehyde gel electrophoresis. Only RNA samples with 260/280 ratio between 1.8 and 2.1 and 260/230 ratio higher than 2.0, as well as both 28S and 18S ribosomal RNA bands with a density ratio about 2∶1 were used for further analyses. Two microgram of total RNA was reverse-transcribed using the ReverTra Ace qPCR RT kit (TOYOBO, Japan) according to the manufacturer’s instructions. The final cDNA products were diluted 150-fold prior to using in RT-qPCR.

### Selection and Cloning of Potential Reference Genes in Papaya

To identify the most stably expressed reference gene(s) to be used in RT-qPCR studies, twenty-one candidate reference genes based on previous reports were selected for investigation in present study. These candidate reference genes included Actin 2 (*ACTIN*), adenine phosphoribosyl transferase (*APT*), cyclophilin (*CYP*), 18S ribosomal RNA (*18S rRNA*), RNA polymerase subunit (*RP*), elongation factor 1-alpha (*EF1*), elongation factor 2-alpha (*EF2*), eukaryotic initiation factor 4A (*EIF*), glyceraldehyde-3-phosphate dehydrogenase (*GAPDH*), GTP-binding nuclear protein (*RAN*), ribosomal protein S (*RPS*), s-adenosyl methionine decarboxylase (*SAMDC*), TATA binding protein 1 (*TBP1*), TATA binding protein 2 (*TBP2*), chymopapain (*CHY*), alpha-tubulin (*TUA*), SAND family protein (*SAND*), ubiquitin conjugating enzyme (*UBCE*), ribulosebisphosphate carboxylase (*RCA*), protein phosphatase 2A regulatory subunit (*PP2A*) and ubiquitin (*UBQ*). Except for *18S rRNA* (GenBank number AY461547.1) and eIF4E (*EIF*) (GenBank number FJ644949.1), *CHY* (GenBank number HQ605970.1) obtained from National Center for Biotechnology Information (NCBI, Bethesda, MD, USA), and *ACTIN*, which cloned by 3′-RACE according to the sequence *Actin* from NCBI (GenBank number FJ696416.1), had large consensus sequence except 3′ tail end, other seventeen candidate reference genes, including *GAPDH, APT, CYP, RAN, EF2, EF1, TBP1, TBP2, SAMDC, TUA, UBQ, RCA, SAND, RP, RPS, PP2A* and *UBCE* were cloned using RT-PCR and RACE-PCR. Degenerate primers were designed using CodeHop Databank (http://bioinformatics.weizmann.ac.il/blocks/codehop.html) within the conserved region of nucleotide sequences aligned by Blockmaker Datebank (http://bioinformatics.weizmann.ac.il/blocks/blockmkr/www/make_blocks.html) from numerous plants found on National Center for Biotechnology Information (NCBI) for PCR amplification. 3′-RACE-PCR was performed using 3′-Full RACE Core Set Ver.2.0 Kit (TaKaRa, Japan).

### Design and Validation of Reference Gene Primers

Primer pairs were designed based on selected sequences of the 21 candidate reference genes using Primer Premier 5.0 and Primer Premier 6.0 software under default parameters. All primers were designed in 3′-untranslated region (3′-UTR), to ensure the specificity of amplification. Ordinarily two or more primer pairs were designed for each gene. Then primers were checked by oligo 6.0 software. All primer pairs were custom-ordered from a commercial supplier (Sangon, Guangzhou, China). Prior to the regular gene expression analysis with RT-qPCR, all primer pairs were tested by RT-qPCR to check for the specificity of the amplicon by the melting-curve after amplification with RT-qPCR analysis. Only primer pairs tested performed well which showed single product and no product in no template control (NTC) were selected for further use. The primer specificities were further confirmed with 2.5% agarose gel (TaKaRa, Japan) electrophoresis and ethidium bromide staining for a single product and the expected size. In addition, the target amplicons were sequenced to confirm specificity of the PCR products. A standard curve using a dilution series of the mixed cDNAs from all tested samples as the template (spanning five orders of magnitude) was made to calculate the gene-specific PCR amplification efficiency (E) and correlation coefficient (R^2^) for each gene. The primer sequences and amplicon characteristics including Tm, length, amplification efficiency and correlation coefficient of the 21 candidate reference genes are listed in Table 2.

**Table 2:** Selected candidate reference genes, primers, and amplicon characteristics.

Gene symbol	Gene name	GenBankaccessionnumeber	Primer sequences(F/R) (5′-3′)	Amplicon length (bp)	Amplicon Tm (°C)	Amplification efficiency (%)	R^2^	
*EF1*	Elongation factor 1-alpha	JQ678770	GGCAGATTGGAAATGGCA AGGAGGATACTGGGAGAA	209	82	98.2		0.999
*EF2*	Elongation factor 2-alpha	JQ678771	CTTTGCCTTCGGTCGTGTCTTC CACTGTCTCCTGCTTCTTTCCC	154	80	94.2		0.998
*RAN*	GTP-binding nuclear protein	JQ678773	GCACAGCAACAGCACGAAG TCACCCCTATCCAAACCAA	199	82	98.1		0.998
*APT*	Adenine phosphoribosyl transferase	JQ678768	TAACCCCTCCAACTAAAAG CCTCGGGAAGTAAACAACT	148	78	91.1		0.992
*CYP*	Cyclophilin	JQ678769	GGAGAGTGGTGGAAGGGATGA GCAGAGCACGGACACAGGAAA	220	84	100		0.998
*GAPDH*	Glyceraldehyde-3-phosphate dehydrogenase	JQ678772	CTTTGTTGGTGACAGCAGG GGACAGAGGCAATGTACC	149	82	99.8		0.998
*TUA*	Alpha-tubulin	JQ678778	TGGTGCTGAAGGTGTGGAA GATCGGAATTGGTTGGGAG	106	78	97.1		0.998
*RP*	RNA polymerase subunit	JQ678774	GAAATCTGGACAAATGGAAG AGGAAAAAAGGGTAAAGTAA	153	77	104.4		0.997
*RPS*	Ribosomal protein S	JQ678775	ACGAAGAAGTTAGAGCCTAC GCAAGTCTGATGTCAATGG	205	84.5	95.2		0.997
*SAMDC*	S-Adenosyl methionine decarboxylase	JQ678781	TAGGTCACTGGAGGAGAAG CAGAGTTGATCTAGGAGAACA	145	81	99.6		0.997
*TBP2*	TATA binding protein 2	JQ678779	TGTGAATACTGGTGCTGAG GGCATGAGACAAGACCTATA	104	80	106.9		0.995
*UBCE*	Ubiquitin conjugating enzyme	JQ678776	GGTCTTTCACCCTAACATC AAATAACCCTTCCTCTCCC	269	82.5	97.8		0.999
*18S rRNA*	18S ribosomal RNA	U42514.1	TCTGCCCGTTGCTCTGATGAT CCTTGGATGTGGTAGCCGTTT	193	84	101.9		0.998
*CHY*	Chymopapain	HQ605970.1	CCAGACAACTTCACTTCAAT CTTCAACAAGGACGCTTA	206	81.5	91.6		0.997
*EIF*	Eukaryotic initiation factor 4A	FJ644949.1	AGGCAGGCAAGAGAAGAT TTCATACCGAGTAGCGATTC	176	81.5	96.7		0.998
*UBQ*	Polyubiquitin	JQ678782	CCTTCTATATGAATGCCTAGC CAGGACATACCAATATCACA	143	76.5	99.4		0.999
*RCA*	Ribulose bisphosphate carboxylase/oxygenase activase	JQ678767	GCAGCTCTTGGAGATGCCAACG TCAACAGAGGCAGCTCCTGTCA	227	80.5	104.9		0.995
*TBP1*	TATA binding protein 2	JQ678780	GGTAGTAGTAGTTAGGTATGTG GGCAATCTGGTCTCACTT	219	79.5	99.6		0.996
*SAND*	Sand family protein	JQ678783	CGTGGTCTGTCAGTGGGTAG ATGATGAGAGGCAAGATGG	246	80	99.1		0.996
*ACTIN*	Actin 2	JQ678785	TTTCCAAGGGTGAGTATGATGAG ACACAGGACACAAAAGCCAACTA	124	79	97.2		0.999
*PP2A*	Protein phosphatase 2A regulatory subunit	JQ678784	CAGTCCCTCGTTCCCATAGT AACAGTGGCATACCTAACTTCC	213	81	100.2		0.998

### RT-qPCR Conditions

RT-qPCR was carried out in 96-well plates with Bio-Rad CFX96 Real-Time PCR System and Bio-Rad CFX96 Manager Software (Bio-Bad, USA) using SYBR Green-based PCR assay. Each reaction mix containing 5 µl diluted cDNAs, 10 µl of THUNDERBIRD SYBR qPCR Mix (TOYOBO, Japan), 0.25 µM of each primer to a final volume of 20 µl was subjected to the following conditions: 95°C for 1 min, followed by 40 cycles of 95°C for 15 s, 55°C for 30 s, and 72°C for 35 s in 96-well optical reaction plates (Bio-Rad, USA). The melting curves were analyzed at 65–95°C after 40 cycles. In addition, reverse transcription negative control was included to check for potential genomic DNA contamination. Each RT-qPCR analysis was performed in triplicate and the mean was used for RT-qPCR analysis.

### Data Analysis

Two publicly available software tools, geNorm (Version3.5) and NormFinder, were used to evaluate the stability of the 21 candidate reference genes under different experimental conditions. The comprehensive ranking of these genes was generated according to a method reported previously [56], [57]. An additional tool, RefFinder (http://www.leonxie.com/referencegene.php), was used to confirm the reliability of calculation. Expression levels of the tested reference genes were determined by CT values [58], the number of amplification threshold cycles needed to reach a specific threshold level of detection. Results were imported into Microsoft Excel and transformed to relative quantities. For each candidate gene, where the highest relative quantity (the minimum CT value) was set to 1, the other average CT value of each duplicate reaction of sample was converted to relative quantity data. Log-transformed data were then exported into geNorm (version 3.5) [15] and NormFinder [31], respectively, to analyze gene expression stability. The geNorm algorithm firstly calculates an expression stability value (M) for each gene and then the pair-wise variation (V) of this gene with the others. All the tested genes are ranked according to their stability in the tested sample sets, and the number of reference genes necessary for an optimal normalization is indicated as well. The NormFinder program identifies the gene(s) with optimal normalization among a set of candidate genes. The lowest stability value indicates the most stable expression within the gene set examined. Therefore, it ranks the set of candidate normalization genes according to the stability of their expression patterns in a given sample set under a given experimental design.

### Normalization of *CpaEXY1*

Endoxylanase acting on matrix polysaccharide xylan degradation is based upon gene expression that occurs during the papaya fruit ripening [51] and plays a role during papaya fruit softening [59]. *CpaEXY1* encoding endoxylanase was obtained from NCBI (Genbank: AY138968.1), used as a target gene to demonstrate the usefulness of the validated candidate reference genes in RT-qPCR. Gene expression levels of *CpaEXY1* were quantified during postharvest ripening using the one or two most stable reference gene(s) and the most unstable gene determined by geNorm and Normfinder in the same RT-qPCR conditions mentioned above. Primer pairs (forward:5′TAATATGGTCAGCGTGGTC3′,reverse:5′GAGATGAGGAAGAAGGTAACT-3′) of *CpaEXY1* were also verified by melting curve analysis and sequencing as described for reference genes.

## Results

### Selection of Candidate Reference Genes and Amplification Specificity

**Figure 1:**
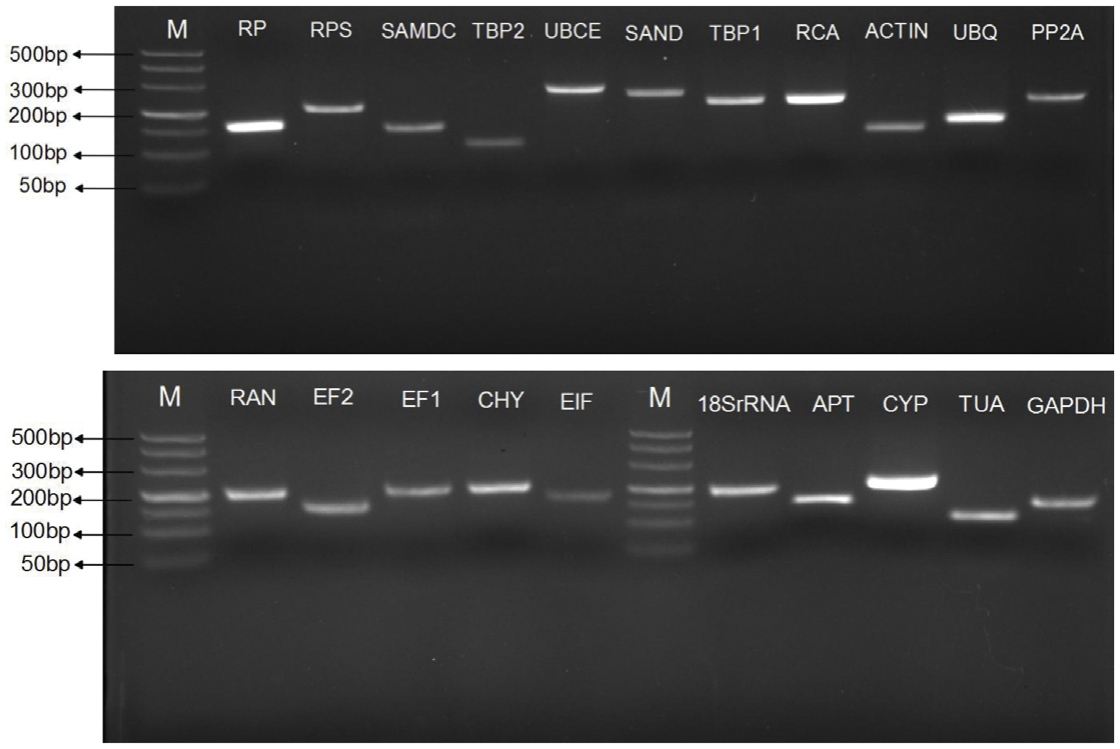
Specificity of primer pairs for RT-qPCR amplification. Equal amounts of cDNAs from all tested samples were mixed as the template. 2.5% non-denaturing agarose gel electrophoresis showed amplification of a specific product of the expected size for each reference gene. M represented DNA size marker.

Twenty-one candidate reference genes based on previous reports were selected and cloned for investigation in present study**.** Primer pairs were designed and selected based on those candidate reference genes. Gene names, accession numbers, the primer sequences and amplicon characteristics including Tm, length, amplification efficiency and correlation coefficient of the 21 candidate reference genes are listed in Table 2. We observed that not all the initially designed primer pairs performed well in the melting curve obtained after 40 cycles of amplification, only those showing single product and no product in no template control (NTC) were selected for further experiment. Specificity of the amplifications was also confirmed by agarose gel electrophoresis, which revealed that the majority of primer pairs selected by melting curve analysis amplified a specific PCR product with the expected size. However, RT-qPCR with the primer pairs for a few candidate reference genes (*ACTIN, UBQ, EF2*) generated multi-products with different size although with the same melting temperature. For these genes, we redesigned and selected and tested additional primer pairs until specific amplifications were detected by both means (Figure 1 and Figure S1). Furthermore, sequence analysis of the cloned amplicons revealed that the amplified fragments were identical or nearly identical (with 1–2 bp mismatched but the sequences of amino acids were fully identical) to the sequences used for primer design. The gene-specific PCR amplification efficiency (E) was calculated by the regression coefficient (R^2^) of the slope of the standard curve. The PCR amplification efficiency for the 21 reference genes varied from 91.1% for *APT* to 106.9% for *TBP2*, and correlation coefficients ranged between 0.992 and 0.999 for *APT* and *EF1*, *UBCE*, *UBQ* or *ACTIN*, respectively (Table 2).

### Expression Profile of the Reference Genes

**Figure 2:**
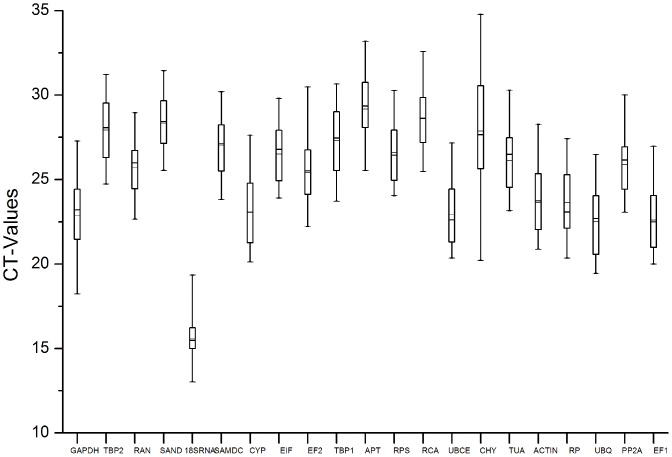
RT-qPCR CT values for the candidate reference genes. Expression data displayed as CT values for each reference gene in all papaya samples. A line across the box is depicted as the median. The box indicates the 25th and 75th percentiles. Whiskers represent the maximum and minimum values.

Some variations amongst 21 reference genes were identified from the analysis of the raw expression levels across all samples (Figure 2). The CT values of these genes ranged from 13.02 to 34.78 in all tested samples, with the majority of these CT values were between 22.51 and 29.18 (Figure 2). The gene encoding *18S rRNA* was highly expressed compared to the protein coding genes, reaching cycle threshold after only 13.02 amplification cycles, whereas the average CT value of all reference genes within the datasets was approximately 25.23 cycles. As a result, the *18S rRNA* transcript levels were about 4100-fold more abundant than the dataset's average. The CT values of *CHY* and *APT* were 34.78 and 33.19, respectively, indicating the least abundant transcripts. The individual reference gene had different expression ranges across all studied samples sets. *SAND* and *EIF* showed smaller gene expression variation (below 6 cycles, with 5.91 and 5.9 cycles, respectively) among studied reference genes, while *GAPDH*, *EF2* and *CHY* had much higher expression variations (above 8 cycles, with 9.06, 8.27 and 14.56 cycles, respectively). The wide expression ranges of the tested reference genes confirmed that no single reference gene has a constant expression in different sets of papaya samples. Therefore, it is of great importance to select the reliable reference gene(s) to normalize gene expression under a certain condition in papaya.

### Expression Stability of Reference Genes

As no one candidate reference gene showed a constant expression in different sets of papaya samples, it was necessary to use statistical methods to rank the stabilities of the 21 genes and to determine the number of reference genes necessary for accurate gene-expression profiling under the given experimental conditions. The programs geNorm and NormFinder, two most widely used algorithms, were used in the following analysis. The RefFinder was used as verification tool.

### GeNorm Analysis

**Figure 3:**
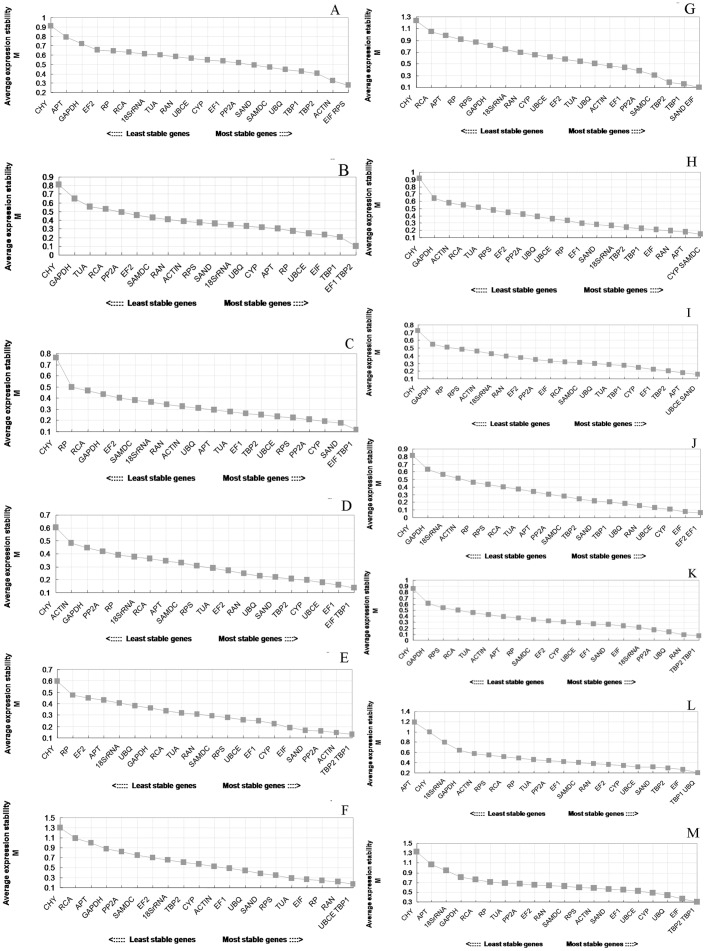
Average expression stability values (M) of the candidate reference genes. Average expression stability values (M) of the reference genes were measured during stepwise exclusion of the least stable reference genes. A lower M value indicated more stable expression, as analyzed by the geNorm software in papaya sample sets under different experimental conditions, including different storage temperatures (a), hot water treatment (b), modified atmosphere packaging (c), hormone treatment (d), 1-MCP fumigation treatment (e), different developmental stages (f), different tissues (g), biotic stress (h), postharvest ripening: cultivar of ‘*Shuiyou 2′* (i), cultivar of ‘*Hongri 1′* (j), cultivar of ‘*Hongri 3′* (k). Different cultivars samples (l) and all papaya samples (m) were also given.

The average expression stability (M) value for each candidate reference gene was calculated based on the average pair-wise variation between all genes tested. The results were presented in Table S1 and Figure 3. Stepwise exclusion of the least stable gene allowed the genes to be ranked according to their M value (the lower the M value, the higher the gene’s expression stability). Among the 21 candidate reference genes used for analysis, not all of the most stable reference genes were identical in the different sample sets (Figure 3 and Table S1). For example, the *EIF* and *RPS* genes were ranked highest in different storage temperatures with an M value of 0.2786 (Figure 3a), whereas the *EF1* and *TBP2* genes were most stably expressed in papaya fruit samples in hot water treatment with an M value of 0.0981 (Figure 3b). For modified atmosphere packaging samples, the most stable genes were *EIF* and *TBP1* with an M value of 0.1181 (Figure 3c), which were the same as the hormone-treated samples but with a different M value of 0.1346 (Figure 3d). The *TBP2* and *TBP1* genes performed best in 1-MCP treatment fruit samples with an M value of 0.1286 (Figure 3e). For the papaya samples at different development stages, the *UBCE* and *TBP1* genes were ranked highest with an M value of 0.1734 (Figure 3f). The *SAND* and *EIF* genes were most stably expressed in different tissue samples with an M value of 0.1030 (Figure 3g). For biotic stress samples, the *CYP* and *SAMDC* genes were proved to be the best with an M value of 0.1508 (Figure 3h). For the postharvest ripening of different papaya cultivar samples, the *UBCE* and *SAND* genes were ranked highest in the cultivars sample of ‘*Shuiyou 2′* with an M value of 0.1596 (Figure 3i), while *EF1* and *EF2* or *TBP1* and *TBP2* in cultivar samples of ‘*Hongri1*’ or ‘*Hongri 3′* were most stably expressed, with an M value of 0.0598 or 0.0777, respectively (Figure 3 j, k). As all the three cultivars samples (for different cultivars) were taken together, the *TBP1* and *UBQ* genes performed best with an M value of 0.2024 (Figure 3l). When all sample sets were analyzed together, the *TBP1* and *TBP2* were the most stably expressed genes with an M value of 0.3056 and might be widely used as a single reference gene for multiple samples (Figure 3m). In contrast, *CHY*, *18S rRNA* and *GAPDH* were the three least stable among the genes examined. These results highlight the importance of choosing the appropriate reference genes according to the experimental conditions.

**Figure 4:**
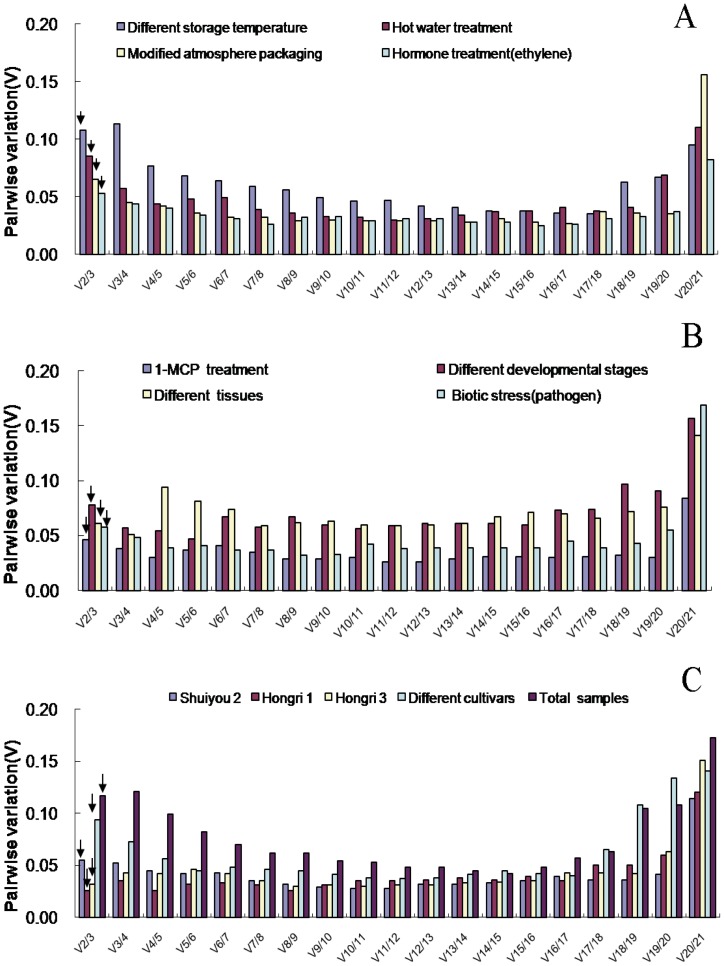
Determination of the optimal number of reference genes. Pair-wise variation (V) calculated by geNorm to determine the minimum number of reference genes for accurate normalization in different experiment conditions. Arrow indicates the optimal number of genes for normalization in each sample sets.

The geNorm program was also applied to calculate the optimal number of reference genes required for accurate normalization in the different sample sets. The software determines the pair-wise variation V_n/n+1_, which measures the effect of adding further reference genes on the normalization factor (that is calculated as the geometric mean of the expression values of the selected reference genes). It is advisable to add additional reference genes to the normalization factor until the pair-wise variation V_n/n+1_ is inferior to a cutoff value (0.15) used by Vandesompele et al. [15], below which the added gene has no significant effect and the inclusion of an additional reference gene is not required. As shown in the Figure 4, pair-wise variation analysis suggested that normalization required the use of only two reference genes in all sample sets since the V_2/3_ value of all sample sets were under the 0.15 cut-off level. Therefore, according to geNorm and NormFinder, the best combinations for all of the sample sets were summarized in Table 3.

**Table 3:** Consensus of stability ranking of the reference gene estimated by geNorm and NormFinder.

Experimental sample sets	The six most stable gene	Most stable combination	The three least stable gene
Different storage temperatures	*EIF RPS SAND TBP2 UBQ ACTIN*	*TBP2+SAND*	*GAPDH APT CHY*
Modified atmosphere packaging	*EIF TBP1 SAND EF1 CYP PP2A*	*EIF+TBP1*	*GAPDH RP CHY*
Hot water treatment	*EF1 TBP2 TBP1 EIF UBCE APT*	*EF1+TBP2*	*TUA GAPDH CHY*
1-MCP treatment	*TBP2 TBP1 ACTIN EIF PP2A CYP*	*TBP2+TBP1*	*APT RP CHY*
Ethephon treatment	*TBP1 EF1 EIF UBCE SAND CYP*	*EF1+TBP1*	*GAPDH ACTIN CHY*
Different development stages	*TBP1 RAN UBCE RP TUA EIF*	*TBP1+RAN*	*APT RCA CHY*
Different tissue	*SAND EIF TBP1 TBP2 SAMDC PP2A*	*SAND+EIF*	*APT RCA CHY*
Biotic stress	*TBP2 CYP SAMDC TBP1 EIF RAN*	*CYP+TBP2*	*ACTIN GAPDH CHY*
*Hongri1*	*EF2 EF1 EIF UBQ CYP UBCE*	*EF2+EF1*	*18S rRNA GAPDH CHY*
*Hongri3*	*TBP2 EIF TBP1 RAN 18S rRNA EF1*	*TBP2+EIF*	*RPS GAPDH CHY*
*Shuiyou2*	*UBCE SAND EF1 APT TBP2 TBP1*	*UBCE+SAND*	*RP GAPDH CHY*
Different cultivars	*TBP1 SAND EIF UBQ SAMDC TBP2*	*TBP1+SAND*	*18S rRNA CHY APT*
Total samples	*EIF TBP1 TBP2 SAND RAN EF1*	*EIF+TBP1*	*18S rRNA APT CHY*

### NormFinder Analysis

NormFinder was also used to evaluate the expression stability of candidate reference genes. More stable gene expression is indicated by lower average expression stability values. In this mathematical model, estimation of both intra- and inter-group variation and a separate analysis of the sample subgroups in expression levels are included into the calculation of a gene expression stability value [16]. Therefore, thirteen sample sets were established as geNorm analysis. At the same time, all samples with no subgroups and the other three sample-subgroups series were analyzed using this approach as well. The results of the Normfinder analysis applied to our data sets were summarized in Table S2. It is noteworthy that definition of sample-subgroups had a notable effect on NormFinder output. However, the NormFinder output with different sample subgroups and no subgroups exhibited almost the same top six stable genes, but with the slight changes in ranking orders. When the outcomes of geNorm and NormFinder were compared, only few differences were observed except for the biotic stress samples, ‘*Hongri 3′* samples and total samples set, which had some obvious nonconformity in the rankings (Table S1, S2).

A method previously described by Chen et al. [56] and Zhang et al. [57] was used to give a comprehensive ranking of candidate reference genes. We firstly assigned a series of continuous integers starting from 1 to 21 as weight to each reference gene, according to the reference genes ranking by each algorithm from the most stable gene to the least stable gene; then we calculated the geometric mean (GM) of each gene weights across the two methods and then re-ranked these reference genes. The gene with the less GM is viewed as more stable reference gene. The comprehensive ranking results were presented in Table S3, and the consensus of the results obtained by both geNorm and NormFinder analysis were summarize in Table 3 according to the performance. In most sample sets, geNorm or NormFinder analysis revealed almost the same top seven stable genes, although with the slight changes in ranking orders and genes. In addition, no matter how the order is changed, the most unstable gene almost remains the same in all sample sets.

### RefFinder Analysis

RefFinder was used to confirm the results obtained from geNorm and NorFinder. RefFinder (http://www.leonxie.com/referencegene.php) is a user-friendly web-based comprehensive tool developed for evaluating and screening reference genes from extensive experimental datasets. It integrates the currently available major computational programs (geNorm, Normfinder, BestKeeper, and the comparative ΔCt method) to compare and rank the tested candidate reference genes. Based on the rankings from each program, it assigns an appropriate weight to an individual gene and calculated the geometric mean of their weights for the overall final ranking. The CT values were input into the program directly and the ranking of the four programs and the comprehensive ranking were then calculated. The data of 1-MCP treatment and modified atmosphere packaging treatment were analyzed by RefFinder and the results were presented in Table S4, which shared a high consistency with the results evaluated by geNorm and Normfinder described above. The top four genes and their ranking were exactly the same, although there was slight difference in the ranking of the other genes. Therefore, these results have proved that the results obtained from the two software, geNorm and Normfinder, were sufficient for our validation.

### Validation for the usefulness of the Selected Reference Gene

**Figure 5:**
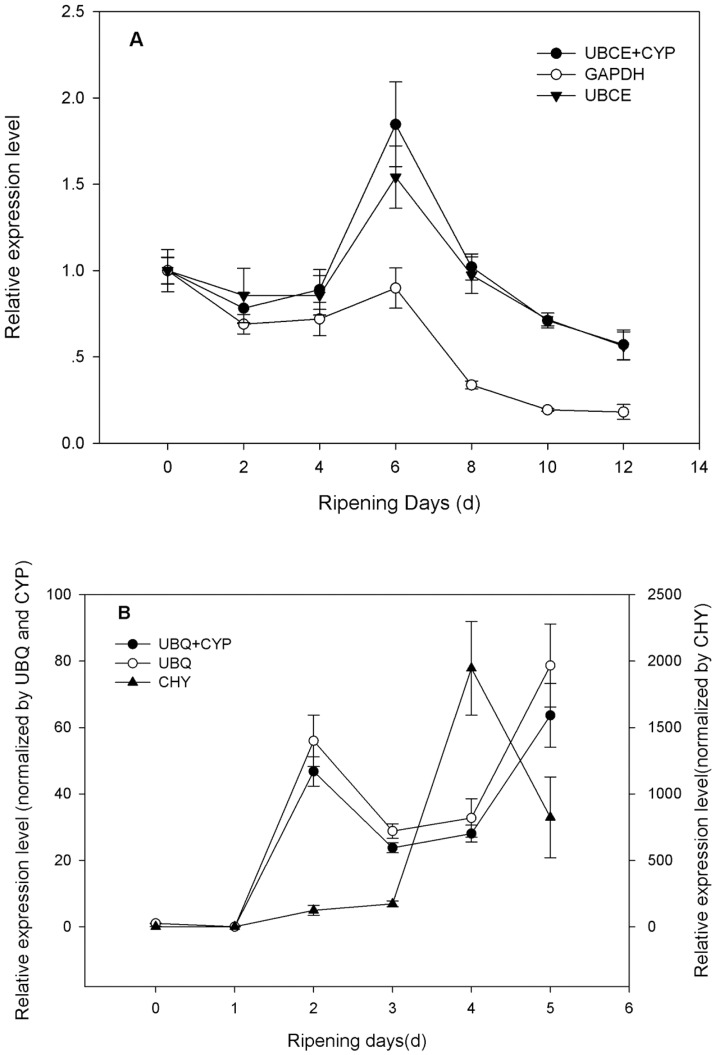
Relative quantification of *CpaEXY1* expression using validated reference genes for normalization under different experimental conditions. (a) The validated reference gene(s) used as normalization factors were one (*UBCE*) or two (*UBCE*+*CYP*) most stable reference genes, and one most unstable gene (*GAPDH*) in postharvest ripening of ‘*Shuiyou 2′* sample sets. (b) The validated reference gene(s) used as normalization factors were one (*UBQ*) or two (*UBQ*+*CYP*) most stable reference genes, and the most unstable one (*CHY*) in postharvest ripening of ‘*Hongri 3′* sample sets. Reference genes validated by geNorm or NormFinder. Each value represented the means of three replicates, and vertical bars indicate the standard deviations (SD). In Figure 5b, *UBQ* +*CYP* and *UBQ* normalized curves belonged to left y axis, and *CHY* normalized curve belonged to right y axis.

It has been documented that the use of inappropriate references can dramatically change the interpretation of the expression pattern of a given target gene [60]. To demonstrate the usefulness of the validated candidate reference genes in RT-qPCR, the relative expression level of one papaya fruit gene, *CpaEXY1,* was investigated in two papaya cultivar fruit during postharvest ripening, using one or two of most stable reference genes, and the most unstable gene for normalization, which had been validated by geNorm or NormFinder as described above (Table S1, 4 and 5; Figure 3). The analysis revealed that the expression level of *CpaEXY1* in ‘*Shuiyou2*’ was not obviously changed in the first four days, but was increased sharply from the 4th to the 6th days, then decreased in the later time. Similar change patterns with slight difference were also seen when *UBCE* alone and the combination of *UBCE*+*CYP* was used as reference gene(s) for normalization, respectively (Figure 5a). *CpaEXY1* expression level in ‘*Hongri1*’ increased from the first two days, then decreased slightly and increased for a second peak during postharvest ripening and also showed similar change patterns when using *CYP* alone and the combination of *CYP* +*UBQ* as reference gene (s) for normalization (Figure 5b). These results were similar to those reported by a former study on *CpaEXY1* gene, with northern-blot analysis [59]. However, these change patterns were completely obscured when the least stable reference gene (*GAPDH*) was used for normalization in ‘*Shuiyou 2′* (Figure 5a) or *CHY* in ‘*Hongri 1′* (Figure 5b). Except for the different expression trends, the expression level of *CpaEXY1* normalized by *CHY* was 25-fold higher than that normalized by *UBQ* or *UBQ*+*CYP* (Figure 5b). This analysis illustrated the adverse effect of using an unsuitable reference gene for normalization and further confirmed the importance of validating reference gene stability to ensure that low precision or misleading results do not occur.

## Discussion

The analysis of gene expression under different experimental conditions is a major aspect of the functional analysis of genes. Currently, one of the most commonly used technologies for gene expression analysis, which can provide more accurate data, is RT-qPCR, a method that combines high specificity and sensitivity [61]. However, quantification of gene expression is affected by several factors, such as the quantity of the initial material, the quality of the RNA, the efficiency of cDNA synthesis, primer performance, and the methods to be used for statistical analysis [38]. Among several normalization strategies that have been proposed, the use of one or more reference genes is currently the preferred way of normalization [6], and it represents a strategy that is simple to use and can control for every stage of the real-time PCR [5]. However, as no single gene has a stable expression under every experimental condition [14], it is advisable to validate the expression stability of candidate reference genes under specific experimental conditions prior to their use in RT-qPCR normalization, rather than using reference genes published elsewhere without validation [62].

Several genes including *GAPDH*, *ACTIN*, *18S rRNA*, *UBQ*, *EF*, *CYP* and *TUA* have been commonly used as the reference genes for gene expression studies in many plant species [12], [29], [36], [38], [63], [64]. However, recent studies have indicated that these traditionally used reference genes are not always stably expressed when tested in other species or in a wider range of experimental treatments [14], [20]. For example, *18S rRNA* and *ACTIN* have been demonstrated to be performed poorly and were less stable during the different treatments [12], [65]. Therefore, it has been stated that the reference genes need to be validated for each plant species and for each specific experimental setup [66]. The results of present study further support this statement. We have demonstrated that *GAPDH* was not the best reference gene but the worst one for normalization during the different treatments in papaya. Furthermore, the most commonly used internal reference genes *ACTIN*, *18S rRNA* and *APT* performed poorly when all the samples were taken together. Unfortunately, almost all current studies on gene expression in papaya fruit with RT-qPCR have used either *Actin* or *18S rRNA* as the reference gene [10], [11], [53], [54]. The poor performance of *Actin* or *18S rRNA* as reference genes indicated the urgent need to identify the other more appropriate reference genes, which serve as the strong reasonable for the present study. To date, a large number of detailed studies have focused on reference gene selection for expression profiles in other kinds of plants [12], [14], [20], [29], [31], [32], [36], [39], [43], [45], [67] but none on papaya. Nevertheless, these studies have provided mass of potential reference genes and allowed the identification of suitable reference gene under a wide range of experimental conditions for papaya possible. In present work, we selected 21 candidate reference genes based on previous reports for identification of the most stably expressed reference gene(s) for normalization under thirteen different sets of experimental conditions in RT-qPCR studies. According to The MIQE Guidelines [58] and the Eleven Golden Rules of Quantitative RT-PCR [68], we tried to control all the sources of variation along the entire workflow of RT-qPCR analysis and used the two most commonly used software, geNorm and NormFinder, to evaluate the expression stability of those candidate reference genes under different experimental conditions. Thus, we conducted a comprehensive evaluation of candidate reference genes in papaya under a wide variety of conditions and treatments. This study allowed identification of the appropriate reference genes suitable for gene expression analyses under different experimental conditions.

It should be pointed out that a single-peak and no product in no template control (NTC) in melting curve analysis do not necessarily mean the single product. Agarose gel electrophoresis should be also performed to confirm the specificity. In this study, we observed that a few selected primer pairs did perform well in melting curves analysis. However, multi-products were detected in the agarose gel electrophoresis analysis. In this case, additional primer pairs needed to be designed and tested until the appropriate primer pairs were found. Especially for the *Actin* gene obtained from NCBI, we hadn’t found an appropriate primer pairs until we cloned a novel gene *Actin 2*, which belong to the *Actin* gene family. As described in the study on reference gene in banana [20], most of the reference genes tested in our study are probably members of large gene families and thus, it is difficult to obtain specific primers.

In the analysis of our datasets, we found that no single reference gene had an optimal performance across all of the experimental conditions tested. The geNorm program ranked *TBP1* and *TBP2* the most stable and best candidates for the normalization of general gene expression for papaya when all papaya samples were tested, and the most commonly used *GAPDH* and *18S rRNA* were proved to be bad reference genes. Different sets of samples had their own best reference genes (Figure 3, Table S1). For example, in the analysis of data with geNorm, reference genes *EIF*, *RPS* and *ACTIN* ranked higher in different storage temperature whereas *EF1*, *TBP2* and *TBP1* did better than *EIF*, *RPS* and *ACTIN* in the hot water treatment. *EIF* and *TBP1* were the best reference genes for MAP and ethephon treatment. For fruit in different development stages, *UBCE* and *TBP1* performed better than others but were not better than *SAND* and *EIF* for different tissues and *CYP* and *SAMDC* for biotic stress samples. All the differences were summarized in Table S1. Our analysis indicated that each experimental condition tested requires a specific set of reference genes. This result emphasizes the importance of reference genes validation for each experimental condition, especially when samples belong to very different sets. This idea is consistent with a number of studies by others [20], [36], [67].

In slight contrast to geNorm, NormFinder ranked *EIF* and *SAND* as the most stably expressed genes in the all samples’ data set, which were also ranked high in the geNorm. For the other sample sets, however, there were some differences observed (Table S1, 4), the ranked high genes almost the same although the ranking orders were somehow different slightly for most of sample sets except of the sets of biotic stress samples, ‘*Hongri 3′* samples and the total samples, which some obviously differences were observed. In addition, no matter how the order was changed, the most unstable gene would almost remain the same in all sample sets, which had been also observed in other studies [14], [20], [31], [60]. Several studies have also reported the similar same results with some minor changes in gene stability ranking [20], [67], [69]. However, others have observed relatively substantial changes between the two methods [32], [60]. GeNorm and NormFinde depend on different mathematical approaches to calculate stability. GeNorm selects two genes with a low intra-group variation and approximately the same non-vanishing inter-group variation. In comparison, NormFinder selects the two best genes with minimal combined inter- and intra-group expression variation [16], which can have a notable effect on the subsequent gene stability ranking [31]. Therefore, the fact that the ranking of candidate reference genes by NormFinder is not always identical to that calculated by geNorm is not surprising.

Taking the two algorithms into account, the consensus of the results obtained by both geNorm and NormFinder analyses were listed in Table S3 and Table 3. From the result obtained by the two programs, *EIF*, *TBP1*, *TBP2* and *EF1* appeared to be suitable as the reference genes for papaya, due to the stability in most of sample sets obtained under different experimental conditions. These results are consistent with those reported on the whole developmental series of tomato for which the *TBP* exhibit a remarkable stability of expression levels [31]. As described in flax [70], both *EF1* and *EIF* genes were the most stable reference genes in flax tissue samples. In contrast, the most commonly used reference gene such as *ACTIN*, *18S rRNA*, *GAPDH*, *TUA* and *APT* were not suitable for most of experimental conditions. Several studies shared the similar results. For example, in petunia, *GAPDH* was considered the least stably expressed gene during leaf and flower development [28]. In tomato, *GAPDH* was poorly ranked as a good reference gene based on the analysis of EST data [30]. *ACT2* was also found to be the least stably expressed gene among the 27 tested in *Arabidopsis* [13]. *18S rRNA* was proved to the least reliable reference gene in peach study under different conditions [12]. *TUA* was found to be not stable for reference gene in the whole developmental series in tomato [31] and in different flax tissues [70], although it showed quite consistent stability in expression in several studies [35], [71]. Thus the use of reference gene should be validated according to the special experimental conditions. On the other hand, considering that the reference gene and the target gene should have a similar range of expression [9], *18S rRNA* might be a good choice as the reference gene for those target genes that have a relative high expression levels under some experimental conditions. However, it should be pointed out that validations of 21 reference genes by the same procedures used in present work do not always give support to their frequent use in other plants, as many studies have suggested that the reference genes are regulated differently in different plant species and might exhibit differential expression patterns [14], [20]. For example, *EIF* or *EF1* show highly stable expression in papaya (present study), *Perennial ryegrass* [67] and banana [20], whereas its putative homologue had been shown unsuitable for normalization in *Petunia hybrida* [28] and tomato [31].

Studies that fail to use appropriate reference genes could lead to bias gene expression profiles and low precision or misleading results [35], [65]. To demonstrate the usefulness of the validated candidate reference genes in RT-qPCR, the relative expression level of *CpaEXY1* was investigated in two papaya cultivar fruits during post-harvest ripening (Figure 5). The results showed that normalization using the most stable reference genes (*UBCE*, *CYP*, *UBQ*) were coincident and similar to those reported in a former study on *CpaEXY1* gene expression by northern-blot analysis [59], but the normalization was obscured when the least stable reference gene(s) (*GAPDH*, *CHY*) were used. These results further confirmed the importance of selection of stable reference genes for the correct normalization of RT-qPCR data.

To the best of our knowledge, this is the first report on the selection of appropriate reference genes using the magnitude of samples tested with different experimental conditions in papaya. Our results provide a foundation for the more accurate and widespread use of RT-qPCR in the analysis of gene expression in papaya. More importantly, our results suggest that *ACTIN*, *18S rRNA* and *GAPDH* are not suitable to be used as reference genes for normalization in papaya under many experimental conditions, whereas *EIF*, *TBP1* and *TBP2* could instead serve well as reference genes due to their good performance in most of experimental conditions. Obviously, the appropriate use of these identified reference genes should be based on the given species and experimental conditions after validation. In addition, using a combination of two genes as reference genes might improve the reliability of gene expression by RT-qPCR in papaya.

## Supporting Information

Figure S1 **Dissociation curve data for the 21 reference genes and one target gene tested.** Dissociation curves for twenty-one candidate reference genes and one target gene *CpaEXY1* showed single peaks and no amplicon was observed in no template control (NTC) indicated by the pink lines.
(DOC)

Table S1 **Candidate genes ranked according to their expression stability value (M) estimated using geNorm algorithm.**
(DOC)

Table S2 **Ranking of candidate reference genes according to their expression stability value calculated by NormFinder.**
(DOC)

Table S3 **Comprehensive ranking of the reference gene estimated by geNorm and NormFinder.**
(DOC)

Table S4 **The results comparison of RefFinder and geNorm + Normfinder.**
(DOC)
