# Supplementary material for: Evaluation of New Reference Genes in Papaya for Accurate Transcript Normalization under Different Experimental Conditions
Source: PLoS One. 2012 Aug 31;7(8):e44405. doi: 10.1371/journal.pone.0044405 (PMC3432124; doi:10.1371/journal.pone.0044405)

**Figure S1** **Dissociation curve data for the 21 reference genes and one target gene tested.** Dissociation curves for twenty-one candidate reference genes and one target gene *CpaEXY1* showed single peaks and no amplicon was observed in no template control (NTC) indicated by the pink lines


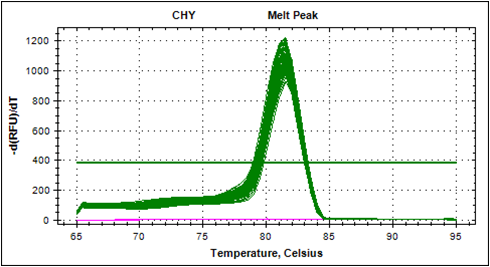

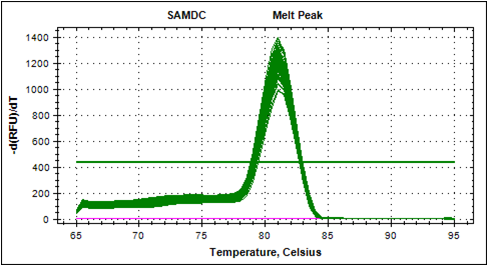

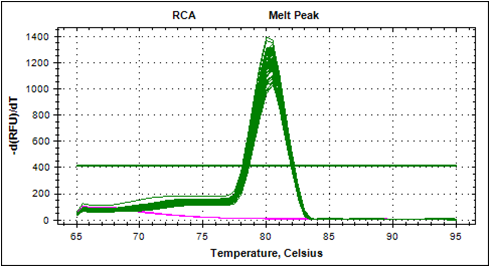

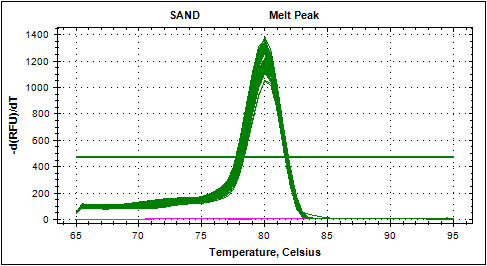

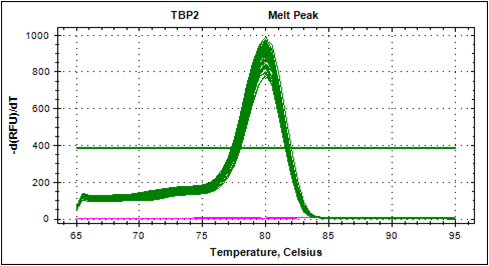

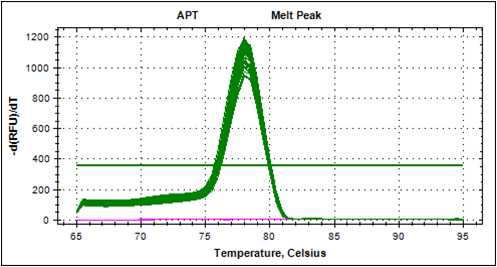

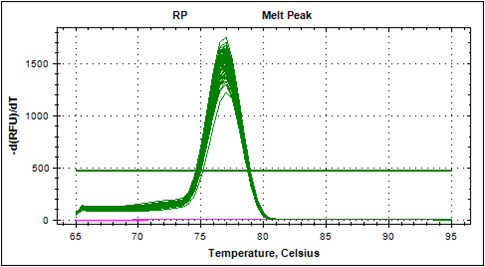

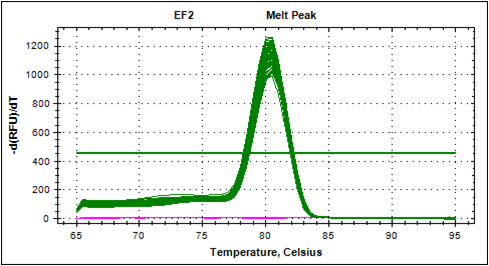

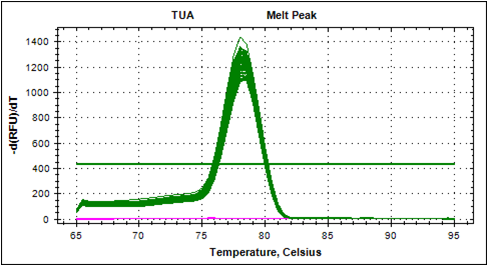

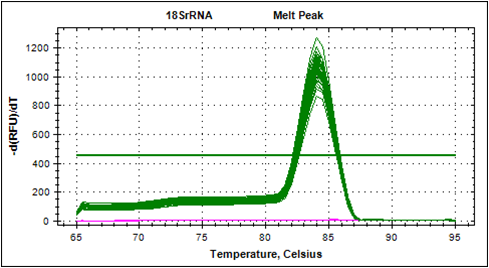

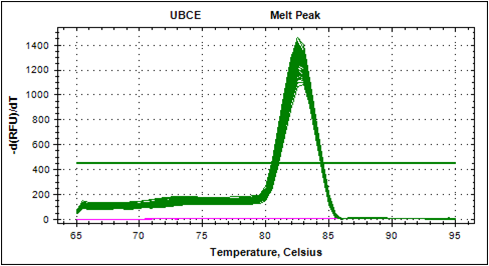

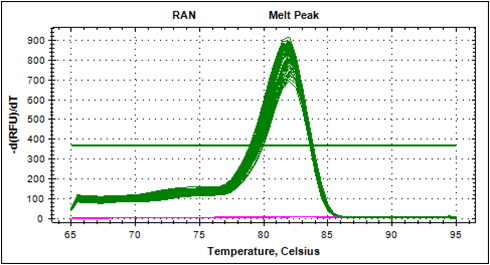

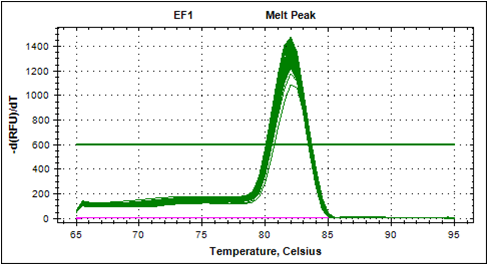

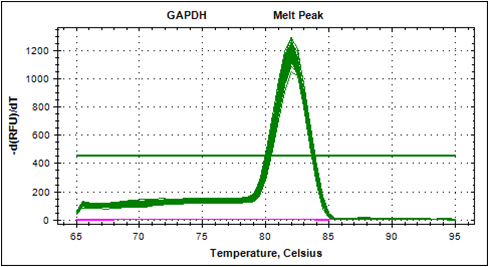

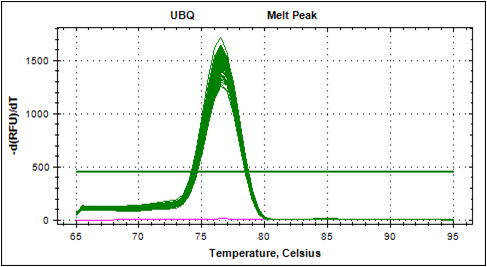

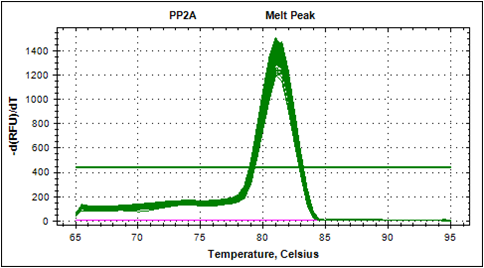

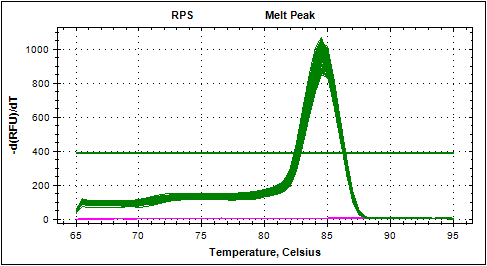

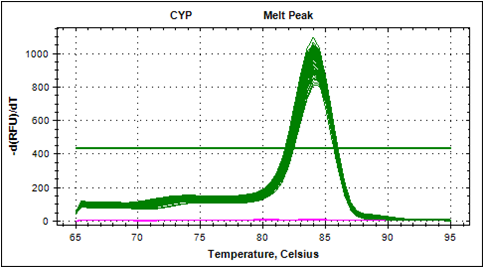

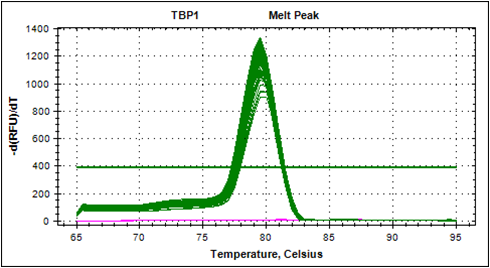

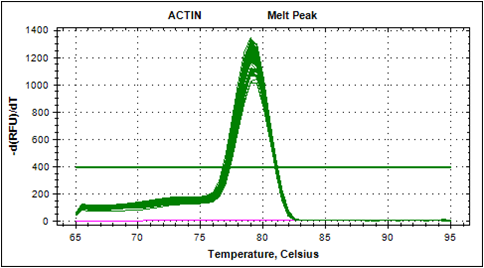

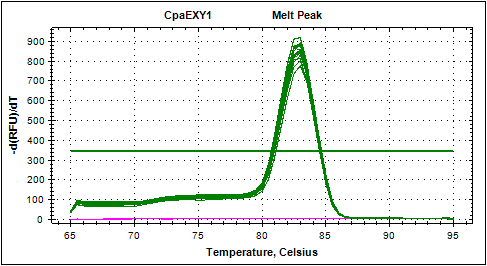

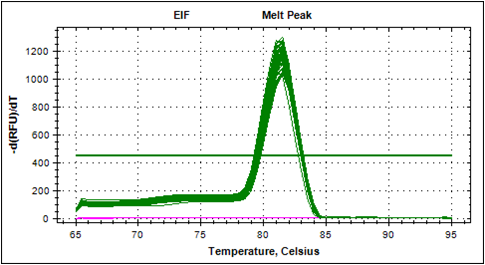

Supplement: Figure S1 — Dissociation curve data for the 21 reference genes and one target gene tested. Dissociation curves for twenty-one candidate reference genes and one target gene CpaEXY1 showed single peaks and no amplicon was observed in no template control (NTC) indicated by the pink lines. (DOC) [file pone.0044405.s001.doc]
