# Supplementary material for: Evaluation of New Reference Genes in Papaya for Accurate Transcript Normalization under Different Experimental Conditions
Source: PLoS One. 2012 Aug 31;7(8):e44405. doi: 10.1371/journal.pone.0044405 (PMC3432124; doi:10.1371/journal.pone.0044405)
Supplement: Table S1 — Candidate genes ranked according to their expression stability value (M) estimated using geNorm algorithm. (DOC) [file pone.0044405.s002.doc]

**Table S1.** Candidates genes ranked according to their expression stability value (M) estimated using geNorm algorithm.

| **Rank** | Different storage temperature | | Modified atmosphere packaging | | Hot water treatment | | 1-MCP treatment | | [Ethephon treatment](app:ds:  ethephon) | | Different development stages | | |
| --- | --- | --- | --- | --- | --- | --- | --- | --- | --- | --- | --- | --- | --- |
| **1** | *EIF RPS* | 0.2786 | *EIF TBP1* | 0.1181 | *EF1 TBP2* | 0.0981 | *TBP2 TBP1* | 0.1286 | *EIF TBP1* | 0.1346 | *UBCE TBP1* | | 0.1734 |
| **2** | *ACTIN* | 0.3248 | *SAND* | 0.1754 | *TBP1* | 0.2063 | *ACTIN* | 0.1445 | *EF1* | 0.1595 | *RAN* | *0.2233* | |
| **3** | *TBP2* | 0.4056 | *CYP* | 0.1910 | *EIF* | 0.2285 | *PP2A* | 0.1593 | *UBCE* | 0.1782 | *RP* | | 0.2414 |
| **4** | *TBP1* | 0.4239 | *PP2A* | 0.2091 | *UBCE* | 0.2435 | *SAND* | 0.1645 | *CYP* | 0.1963 | *EIF* | | 0.2637 |
| **5** | *UBQ* | 0.4458 | *RPS* | 0.2233 | *RP* | 0.2722 | *EIF* | 0.1905 | *TBP2* | 0.2082 | *TUA* | | 0.2825 |
| **6** | *SAMDC* | 0.4702 | *UBCE* | 0.2350 | *APT* | 0.3023 | *CYP* | 0.2226 | *SAND* | 0.2207 | *RPS* | | 0.3422 |
| **7** | *SAND* | 0.4918 | *TBP2* | 0.2497 | *CYP* | 0.3168 | *EF1* | 0.2454 | *UBQ* | 0.2288 | *SAND* | | 0.3855 |
| **8** | *PP2A* | 0.5137 | *EF1* | 0.2617 | *UBQ* | 0.3312 | *UBCE* | 0.2587 | *RAN* | 0.2486 | *UBQ* | | 0.4420 |
| **9** | *EF1* | 0.5317 | *TUA* | 0.2757 | *18SrRNA* | 0.3440 | *RPS* | 0.2746 | *EF2* | 0.2721 | *EF1* | | 0.4873 |
| **10** | *CYP* | 0.5466 | *APT* | 0.2928 | *SAND* | 0.3583 | *SAMDC* | 0.2926 | *TUA* | 0.2888 | *ACTIN* | | 0.5230 |
| **11** | *UBCE* | 0.5669 | *UBQ* | 0.3095 | *RPS* | 0.3695 | *RAN* | 0.3043 | *RPS* | 0.3093 | *CYP* | | 0.5658 |
| **12** | *RAN* | 0.5830 | *ACTIN* | 0.3279 | *ACTIN* | 0.3844 | *TUA* | 0.3180 | *SAMDC* | 0.3307 | *TBP2* | | 0.6107 |
| **13** | *TUA* | 0.5983 | *RAN* | 0.3438 | *RAN* | 0.4052 | *RCA* | 0.3379 | *APT* | 0.3467 | *18SrRNA* | | 0.6580 |
| **14** | *18SrRNA* | 0.6125 | *18SrRNA* | 0.3655 | *SAMDC* | 0.4310 | *GAPDH* | 0.3595 | *RCA* | 0.3644 | *EF2* | | 0.7030 |
| **15** | *RCA* | 0.6276 | *SAMDC* | 0.3826 | *EF2* | 0.4596 | *UBQ* | 0.3818 | *18SrRNA* | 0.3776 | *SAMDC* | | 0.7465 |
| **16** | *RP* | 0.6424 | *EF2* | 0.3989 | *PP2A* | 0.4956 | *18SrRNA* | 0.4033 | *RP* | 0.3930 | *PP2A* | | 0.8137 |
| **17** | *EF2* | 0.6554 | *GAPDH* | 0.4332 | *RCA* | 0.5248 | *APT* | 0.4271 | *PP2A* | 0.4174 | *GAPDH* | | 0.8823 |
| **18** | *GAPDH* | 0.7193 | *RCA* | 0.4652 | *TUA* | 0.5584 | *EF2* | 0.4507 | *GAPDH* | 0.4458 | *APT* | | 0.9909 |
| **19** | *APT* | 0.7894 | *RP* | 0.4960 | *GAPDH* | 0.6442 | *RP* | 0.4727 | *ACTIN* | 0.4808 | *RCA* | | 1.0837 |
| **20** | *CHY* | 0.9109 | *CHY* | 0.7615 | *CHY* | 0.8054 | *CHY* | 0.5981 | *CHY* | 0.6025 | *CHY* | | 1.3028 |

Continual

| **Rank** | Different tissue | | Biotic stress | | *Hongri1* | | *Hongri3* | | *Shuiyou 2* | | Different cultivars | | Total samples | |
| --- | --- | --- | --- | --- | --- | --- | --- | --- | --- | --- | --- | --- | --- | --- |
| 1 | *SAND EIF* | 0.1030 | *CYP SAMDC* | 0.1508 | *EF2 EF1* | 0.0598 | *TBP2 TBP1* | 0.0777 | *UBCE SAND* | 0.1596 | *TBP1 UBQ* | 0.2024 | *TBP2 TBP1* | 0.3056 |
| 2 | *TBP1* | 0.1604 | *APT* | 0.1774 | *EIF* | 0.0744 | *RAN* | 0.0939 | *APT* | 0.1759 | *EIF* | 0.2656 | *EIF* | 0.3579 |
| 3 | *TBP2* | 0.1878 | *RAN* | 0.1970 | *CYP* | 0.1095 | *UBQ* | 0.1373 | *TBP2* | 0.2033 | *TBP2* | 0.2969 | *UBQ* | 0.4415 |
| 4 | *SAMDC* | 0.3056 | *EIF* | 0.2085 | *UBCE* | 0.1253 | *PP2A* | 0.1738 | *EF1* | 0.2239 | *SAND* | 0.3119 | *CYP* | 0.4916 |
| 5 | *PP2A* | 0.3806 | *TBP1* | 0.2280 | *RAN* | 0.1523 | *18SrRNA* | 0.2119 | *CYP* | 0.2441 | *UBCE* | 0.3194 | *UBCE* | 0.5233 |
| 6 | *EF1* | 0.4359 | *TBP2* | 0.2434 | *UBQ* | 0.1812 | *EIF* | 0.2411 | *TBP1* | 0.2696 | *CYP* | 0.3406 | *EF1* | 0.5427 |
| 7 | *ACTIN* | 0.4612 | *18SrRNA* | 0.2650 | *TBP1* | 0.2042 | *SAND* | 0.2606 | *TUA* | 0.2852 | *EF2* | 0.3608 | *SAND* | 0.5586 |
| 8 | *UBQ* | 0.4990 | *SAND* | 0.2801 | *SAND* | 0.2195 | *EF1* | 0.2730 | *UBQ* | 0.2962 | *RAN* | 0.3842 | *ACTIN* | 0.5827 |
| 9 | *TUA* | 0.5414 | *EF1* | 0.2974 | *TBP2* | 0.2437 | *UBCE* | 0.2900 | *SAMDC* | 0.3077 | *SAMDC* | 0.4029 | *RPS* | 0.5993 |
| 10 | *EF2* | 0.5781 | *RP* | 0.3337 | *SAMDC* | 0.2750 | *CYP* | 0.3048 | *RCA* | 0.3183 | *EF1* | 0.4198 | *SAMDC* | 0.6179 |
| 11 | *UBCE* | 0.6147 | *UBCE* | 0.3611 | *PP2A* | 0.3048 | *EF2* | 0.3226 | *EIF* | 0.3301 | *PP2A* | 0.4368 | *RAN* | 0.6329 |
| 12 | *CYP* | 0.6538 | *UBQ* | 0.3901 | *APT* | 0.3359 | *SAMDC* | 0.3426 | *PP2A* | 0.3501 | *TUA* | 0.4579 | *EF2* | 0.6523 |
| 13 | *RAN* | 0.6951 | *PP2A* | 0.4177 | *TUA* | 0.3701 | *RP* | 0.3679 | *EF2* | 0.3711 | *RP* | 0.4862 | *PP2A* | 0.6687 |
| 14 | *18SrRNA* | 0.7471 | *EF2* | 0.4471 | *RCA* | 0.3995 | *APT* | 0.3941 | *RAN* | 0.3952 | *RCA* | 0.5199 | *TUA* | 0.6830 |
| 15 | *GAPDH* | 0.8082 | *RPS* | 0.4774 | *RPS* | 0.4308 | *ACTIN* | 0.4222 | *18SrRNA* | 0.4232 | *RPS* | 0.5488 | *RP* | 0.7101 |
| 16 | *RPS* | 0.8661 | *TUA* | 0.5158 | *RP* | 0.4583 | *TUA* | 0.4620 | *ACTIN* | 0.4570 | *ACTIN* | 0.5743 | *RCA* | 0.7533 |
| 17 | *RP* | 0.9174 | *RCA* | 0.5453 | *ACTIN* | 0.5112 | *RCA* | 0.5026 | *RPS* | 0.4829 | *GAPDH* | 0.6460 | *GAPDH* | 0.8072 |
| 18 | *APT* | 0.9786 | *ACTIN* | 0.5801 | *18SrRNA* | 0.5640 | *RPS* | 0.5389 | *RP* | 0.5097 | *18SrRNA* | 0.7991 | *18SrRNA* | 0.9399 |
| 19 | *RCA* | 1.0467 | *GAPDH* | 0.6363 | *GAPDH* | 0.6324 | *GAPDH* | 0.6137 | *GAPDH* | 0.5460 | *CHY* | 0.9936 | *APT* | 1.0724 |
| 20 | *CHY* | 1.2369 | *CHY* | 0.9159 | *CHY* | 0.8136 | *CHY* | 0.8596 | *CHY* | 0.7242 | *APT* | 1.1904 | *CHY* | 1.3250 |
