# Supplementary material for: Evaluation of New Reference Genes in Papaya for Accurate Transcript Normalization under Different Experimental Conditions
Source: PLoS One. 2012 Aug 31;7(8):e44405. doi: 10.1371/journal.pone.0044405 (PMC3432124; doi:10.1371/journal.pone.0044405)
Supplement: Table S2 — Ranking of candidate reference genes according to their expression stability value calculated by NormFinder. (DOC) [file pone.0044405.s003.doc]

**Table S2.** Ranking of candidate reference genes according to their expression stability value calculated by NormFinder

| Rank | Modified atmosphere packaging | | Hot water treatment | | 1-MCP treatment | | [Ethephon treatment](app:ds:  ethephon) | | Biotic stress | | Different development stages | | Different tissue | | *Hongri 1* |  | *Hongri 3* |  |
| --- | --- | --- | --- | --- | --- | --- | --- | --- | --- | --- | --- | --- | --- | --- | --- | --- | --- | --- |
| 1 | *EIF* | 0.0409 | *EF1* | 0.0340 | *TBP2* | 0.0446 | *EF1* | 0.0787 | *TBP2* | 0.0424 | *TBP1* | 0.0576 | *SAND* | 0.0357 | *UBQ* | 0.0093 | *EIF* | 0.0321 |
| 2 | *EF1* | 0.0791 | *TBP2* | 0.0340 | *EIF* | 0.0529 | *TBP1* | 0.0816 | *TBP1* | 0.0424 | *RAN* | 0.0708 | *EIF* | 0.0357 | *EF2* | 0.0207 | *18SrRNA* | 0.0568 |
| 3 | *TBP1* | 0.0914 | *TBP1* | 0.1056 | *TBP1* | 0.0984 | *EIF* | 0.1043 | *EIF* | 0.0556 | *RP* | 0.0708 | *TBP1* | 0.1151 | *EIF* | 0.0209 | *EF1* | 0.0607 |
| 4 | *SAND* | 0.1053 | *EIF* | 0.1505 | *ACTIN* | 0.1165 | *TUA* | 0.1158 | *18SrRNA* | 0.0631 | *TUA* | 0.1328 | *TBP2* | 0.1576 | *EF1* | 0.0399 | *SAND* | 0.0653 |
| 5 | *SAMDC* | 0.1433 | *APT* | 0.1636 | *CYP* | 0.1277 | *SAND* | 0.1165 | *EF1* | 0.0631 | *UBCE* | 0.1481 | *UBQ* | 0.3445 | *CYP* | 0.0433 | *RAN* | 0.0936 |
| 6 | *PP2A* | 0.1482 | *CYP* | 0.1726 | *UBCE* | 0.1438 | *EF2* | 0.1424 | *RAN* | 0.1217 | *EIF* | 0.1663 | *SAMDC* | 0.3480 | *UBCE* | 0.0587 | *TBP2* | 0.1320 |
| 7 | *RAN* | 0.1586 | *UBCE* | 0.1742 | *EF1* | 0.1467 | *UBCE* | 0.1461 | *CYP* | 0.1705 | *SAND* | 0.2095 | *PP2A* | 0.4540 | *SAND* | 0.0716 | *EF2* | 0.1403 |
| 8 | *CYP* | 0.1794 | *RP* | 0.1809 | *SAND* | 0.1561 | *CYP* | 0.1978 | *SAMDC* | 0.1956 | *RPS* | 0.3201 | *EF1* | 0.4634 | *RAN* | 0.0977 | *SAMDC* | 0.1800 |
| 9 | *ACTIN* | 0.2042 | *ACTIN* | 0.2163 | *PP2A* | 0.1575 | *RAN* | 0.2067 | *SAND* | 0.2036 | *TBP2* | 0.3472 | *ACTIN* | 0.4856 | *TBP1* | 0.1463 | *TBP1* | 0.1849 |
| 10 | *TBP2* | 0.2052 | *UBQ* | 0.2327 | *RPS* | 0.1623 | *RPS* | 0.2134 | *UBCE* | 0.2101 | *EF1* | 0.4975 | *UBCE* | 0.4963 | *TBP2* | 0.2220 | *UBCE* | 0.2039 |
| 11 | *UBCE* | 0.2390 | *RAN* | 0.2683 | *SAMDC* | 0.1967 | *UBQ* | 0.2136 | *RP* | 0.2203 | *UBQ* | 0.5129 | *18SrRNA* | 0.5097 | *SAMDC* | 0.2764 | *PP2A* | 0.2414 |
| 12 | *APT* | 0.2420 | *18SrRNA* | 0.2844 | *TUA* | 0.2049 | *TBP2* | 0.2181 | *APT* | 0.2256 | *SAMDC* | 0.5215 | *RAN* | 0.5331 | *PP2A* | 0.3097 | *UBQ* | 0.2569 |
| 13 | *18SrRNA* | 0.2618 | *SAND* | 0.2894 | *RAN* | 0.2203 | *18SrRNA* | 0.2414 | *EF2* | 0.3207 | *ACTIN* | 0.5306 | *TUA* | 0.5932 | *RPS* | 0.3131 | *ACTIN* | 0.3097 |
| 14 | *RPS* | 0.2644 | *RPS* | 0.3153 | *RCA* | 0.2586 | *SAMDC* | 0.2455 | *UBQ* | 0.3472 | *18SrRNA* | 0.5836 | *EF2* | 0.5979 | *RP* | 0.3630 | *CYP* | 0.3197 |
| 15 | *EF2* | 0.2705 | *SAMDC* | 0.3462 | *GAPDH* | 0.3312 | *RCA* | 0.2478 | *RPS* | 0.3847 | *CYP* | 0.6805 | *CYP* | 0.6005 | *TUA* | 0.3833 | *RP* | 0.3357 |
| 16 | *TUA* | 0.3271 | *EF2* | 0.3557 | *EF2* | 0.3701 | *APT* | 0.2574 | *PP2A* | 0.4159 | *EF2* | 0.7986 | *RP* | 0.7185 | *APT* | 0.3953 | *APT* | 0.3426 |
| 17 | *UBQ* | 0.3587 | *RCA* | 0.4300 | *UBQ* | 0.3719 | *RP* | 0.2989 | *ACTIN* | 0.5142 | *GAPDH* | 0.8822 | *GAPDH* | 0.7717 | *RCA* | 0.4531 | *RPS* | 0.4705 |
| 18 | *RCA* | 0.3644 | *PP2A* | 0.4563 | *APT* | 0.3778 | *PP2A* | 0.4238 | *RCA* | 0.5474 | *PP2A* | 0.9748 | *RPS* | 0.7825 | *ACTIN* | 0.5575 | *RCA* | 0.4726 |
| 19 | *RP* | 0.4371 | *TUA* | 0.5552 | *18SrRNA* | 0.4021 | *ACTIN* | 0.4784 | *TUA* | 0.5779 | *APT* | 1.1335 | *APT* | 0.8885 | *18SrRNA* | 0.6506 | *TUA* | 0.5578 |
| 20 | *GAPDH* | 0.5522 | *GAPDH* | 1.0210 | *RP* | 0.4653 | *GAPDH* | 0.4879 | *GAPDH* | 0.8714 | *RCA* | 1.2151 | *RCA* | 0.9832 | *GAPDH* | 0.9071 | *GAPDH* | 0.9627 |
| 21 | *CHY* | 2.2660 | *CHY* | 1.6025 | *CHY* | 1.2261 | *CHY* | 1.1988 | *CHY* | 2.4652 | *CHY* | 2.2834 | *CHY* | 2.0436 | *CHY* | 1.7405 | *CHY* | 2.2029 |

Continual

| Rank | *Shuiyou 2* |  | Different cultivars | |  |  | Different storage temperature | | |  | | Total samples | | | |
| --- | --- | --- | --- | --- | --- | --- | --- | --- | --- | --- | --- | --- | --- | --- | --- |
|  |  |  | No subgroups | | 3 subgroups |  | No subgroups | | 4 subgroups | | | No subgroups | | 8 subgroups |  |
| 1 | *UBCE* | 0.0439 | *SAND* | 0.1470 | *RAN* | 0.1425 | *EIF* | 0.1275 | *EIF* | | 0.1205 | *EIF* | 0.1610 | *EIF* | 0.1931 |
| 2 | *EF1* | 0.0711 | *SAMDC* | 0.1633 | *SAMDC* | 0.1718 | *SAND* | 0.1926 | *RPS* | | 0.1593 | *SAND* | 0.2041 | *RAN* | 0.2193 |
| 3 | *TBP1* | 0.1114 | *EIF* | 0.1704 | *PP2A* | 0.1839 | *UBQ* | 0.2004 | *UBQ* | | 0.1721 | *RAN* | 0.2775 | *TBP1* | 0.2424 |
| 4 | *SAND* | 0.1273 | *TBP1* | 0.1822 | *SAND* | 0.1971 | *RPS* | 0.2327 | *SAND* | | 0.2121 | *SAMDC* | 0.2884 | *TBP2* | 0.2511 |
| 5 | *CYP* | 0.1286 | *RAN* | 0.1948 | *EIF* | 0.1988 | *TBP2* | 0.2530 | *PP2A* | | 0.2438 | *TBP2* | 0.2980 | *SAND* | 0.2642 |
| 6 | *TBP2* | 0.1430 | *UBCE* | 0.2104 | *TBP1* | 0.2237 | *EF1* | 0.2593 | *TBP2* | | 0.2571 | *EF1* | 0.3276 | *SAMDC* | 0.2943 |
| 7 | *SAMDC* | 0.1546 | *EF2* | 0.2432 | *UBCE* | 0.2308 | *PP2A* | 0.2732 | *CYP* | | 0.2575 | *TBP1* | 0.3390 | *EF1* | 0.3087 |
| 8 | *UBQ* | 0.1565 | *PP2A* | 0.2481 | *EF2* | 0.2415 | *CYP* | 0.2964 | *ACTIN* | | 0.2616 | *UBCE* | 0.3563 | *TUA* | 0.3571 |
| 9 | *APT* | 0.1719 | *UBQ* | 0.2492 | *UBQ* | 0.2697 | *ACTIN* | 0.3001 | *EF1* | | 0.2773 | *PP2A* | 0.3907 | *UBCE* | 0.3591 |
| 10 | *RCA* | 0.1818 | *TBP2* | 0.2725 | *RCA* | 0.2771 | *SAMDC* | 0.3051 | *SAMDC* | | 0.2916 | *RPS* | 0.3970 | *PP2A* | 0.3719 |
| 11 | *EIF* | 0.1965 | *RCA* | 0.3477 | *TBP2* | 0.3056 | *TBP1* | 0.3493 | *18SrRNA* | | 0.3012 | *TUA* | 0.4142 | *EF2* | 0.3810 |
| 12 | *TUA* | 0.2153 | *RPS* | 0.3921 | *RPS* | 0.3482 | *TUA* | 0.3540 | *RAN* | | 0.3053 | *EF2* | 0.4212 | *ACTIN* | 0.3879 |
| 13 | *PP2A* | 0.3235 | *CYP* | 0.4021 | *EF1* | 0.3602 | *18SrRNA* | 0.3739 | *RP* | | 0.3285 | *UBQ* | 0.4221 | *RPS* | 0.3884 |
| 14 | *EF2* | 0.3352 | *EF1* | 0.4060 | *CYP* | 0.4141 | *UBCE* | 0.3793 | *TUA* | | 0.3286 | *CYP* | 0.4336 | *CYP* | 0.3927 |
| 15 | *18SrRNA* | 0.3706 | *TUA* | 0.4426 | *TUA* | 0.4282 | *RAN* | 0.3889 | *EF2* | | 0.3539 | *ACTIN* | 0.4607 | *UBQ* | 0.3993 |
| 16 | *RAN* | 0.3767 | *RP* | 0.4713 | *RP* | 0.4485 | *RP* | 0.4249 | *TBP1* | | 0.3547 | *RP* | 0.5229 | *RP* | 0.5177 |
| 17 | *ACTIN* | 0.3791 | *ACTIN* | 0.4806 | *ACTIN* | 0.4579 | *EF2* | 0.4346 | *UBCE* | | 0.3561 | *RCA* | 0.5892 | *RCA* | 0.5306 |
| 18 | *RPS* | 0.3956 | *GAPDH* | 0.9583 | *GAPDH* | 0.8265 | *RCA* | 0.4571 | *RCA* | | 0.4532 | *GAPDH* | 0.8251 | *GAPDH* | 0.6187 |
| 19 | *RP* | 0.4928 | *18SrRNA* | 1.2816 | *CHY* | 1.0737 | *GAPDH* | 0.8358 | *APT* | | 0.6297 | *18SrRNA* | 1.3208 | *APT* | 0.8624 |
| 20 | *GAPDH* | 0.6398 | *CHY* | 1.8282 | *18SrRNA* | 1.1791 | *APT* | 0.9415 | *GAPDH* | | 0.7061 | *APT* | 1.4912 | *18SrRNA* | 1.2853 |
| 21 | *CHY* | 1.6638 | *APT* | 2.0533 | *APT* | 1.8511 | *CHY* | 1.3823 | *CHY* | | 0.7318 | *CHY* | 2.5170 | *CHY* | 1.7775 |
|  | Best combination | |  | *RAN* and *PP2A* | | 0.1273 |  | *TBP2* and *SAND* | | | 0.0945 |  | *RAN* and *UBCE* | | 0.1725 |
