# Supplementary material for: Evaluation of New Reference Genes in Papaya for Accurate Transcript Normalization under Different Experimental Conditions
Source: PLoS One. 2012 Aug 31;7(8):e44405. doi: 10.1371/journal.pone.0044405 (PMC3432124; doi:10.1371/journal.pone.0044405)
Supplement: Table S3 — Comprehensive ranking of the reference gene estimated by geNorm and NormFinder. (DOC) [file pone.0044405.s004.doc]

**Table S3**.Comprehensive ranking of the reference gene estimated by geNorm and NormFinder

| Rank | Different stored temperature | Modified atmosphere packaging | Hot water treatment | 1-MCP treatment | [Ethephon treatment](app:ds:  ethephon) | Different development stages | Different tissue | Biotic stress | *Hongri 1* | *Hongri 3* | *Shuiyou 2* | Different cultivars | Total samples |
| --- | --- | --- | --- | --- | --- | --- | --- | --- | --- | --- | --- | --- | --- |
| 1 | *EIF* | *EIF* | *EF1* | *TBP2* | *TBP1* | *TBP1* | *SAND* | *TBP2* | *EF2* | *TBP2* | *UBCE* | *TBP1* | *EIF* |
| 2 | *RPS* | *TBP1* | *TBP2* | *TBP1* | *EF1* | *RAN* | *EIF* | *CYP* | *EF1* | *EIF* | *SAND* | *SAND* | *TBP2* |
| 3 | *SAND* | *SAND* | *TBP1* | *ACTIN* | *EIF* | *UBCE* | *TBP1* | *SAMDC* | *EIF* | *TBP1* | *EF1* | *EIF* | *TBP1* |
| 4 | *TBP2* | *EF1* | *EIF* | *EIF* | *UBCE* | *RP* | *TBP2* | *TBP1* | *UBQ* | *RAN* | *APT* | *UBQ* | *SAND* |
| 5 | *UBQ* | *CYP* | *UBCE* | *PP2A* | *SAND* | *TUA* | *SAMDC* | *EIF* | *CYP* | *18SrRNA* | *TBP2* | *SAMDC* | *RAN* |
| 6 | *ACTIN* | *PP2A* | *APT* | *CYP* | *CYP* | *EIF* | *PP2A* | *RAN* | *UBCE* | *EF1* | *TBP1* | *TBP2* | *EF1* |
| 7 | *TBP1* | *UBCE* | *RP* | *SAND* | *TUA* | *RPS* | *UBQ* | *APT* | *RAN* | *SAND* | *CYP* | *UBCE* | *UBQ* |
| 8 | *EF1* | *RPS* | *CYP* | *UBCE* | *EF2* | *SAND* | *EF1* | *18SrRNA* | *SAND* | *UBQ* | *SAMDC* | *RAN* | *UBCE* |
| 9 | *PP2A* | *TBP2* | *UBQ* | *EF1* | *TBP2* | *UBQ* | *ACTIN* | *EF1* | *TBP1* | *PP2A* | *UBQ* | *EF2* | *SAMDC* |
| 10 | *SAMDC* | *SAMDC* | *18SrRNA* | *RPS* | *RAN* | *EF1* | *UBCE* | *SAND* | *TBP2* | *EF2* | *TUA* | *CYP* | *CYP* |
| 11 | *CYP* | *RAN* | *ACTIN* | *SAMDC* | *UBQ* | *TBP2* | *TUA* | *RP* | *SAMDC* | *UBCE* | *RCA* | *PP2A* | *RPS* |
| 12 | *UBCE* | *ACTIN* | *SAND* | *RAN* | *RPS* | *ACTIN* | *EF2* | *UBCE* | *PP2A* | *SAMDC* | *EIF* | *EF1* | *PP2A* |
| 13 | *TUA* | *APT* | *RAN* | *TUA* | *SAMDC* | *CYP* | *18SrRNA* | *UBQ* | *APT* | *CYP* | *PP2A* | *RCA* | *ACTIN* |
| 14 | *RAN* | *TUA* | *RPS* | *RCA* | *18SrRNA* | *SAMDC* | *RAN* | *EF2* | *TUA* | *RP* | *EF2* | *TUA* | *EF2* |
| 15 | *18SrRNA* | *18SrRNA* | *SAMDC* | *GAPDH* | *APT* | *18SrRNA* | *CYP* | *PP2A* | *RPS* | *ACTIN* | *RAN* | *RPS* | *TUA* |
| 16 | *RP* | *UBQ* | *EF2* | *UBQ* | *RCA* | *EF2* | *GAPDH* | *RPS* | *RP* | *APT* | *18SrRNA* | *RP* | *RP* |
| 17 | *RCA* | *EF2* | *PP2A* | *EF2* | *RP* | *PP2A* | *RP* | *TUA* | *RCA* | *TUA* | *ACTIN* | *ACTIN* | *RCA* |
| 18 | *EF2* | *RCA* | *RCA* | *18SrRNA* | *PP2A* | *GAPDH* | *RPS* | *RCA* | *ACTIN* | *RCA* | *RPS* | *GAPDH* | *GAPDH* |
| 19 | *GAPDH* | *GAPDH* | *TUA* | *APT* | *GAPDH* | *APT* | *APT* | *ACTIN* | *18SrRNA* | *RPS* | *RP* | *18SrRNA* | *18SrRNA* |
| 20 | *APT* | *RP* | *GAPDH* | *RP* | *ACTIN* | *RCA* | *RCA* | *GAPDH* | *GAPDH* | *GAPDH* | *GAPDH* | *CHY* | *APT* |
| 21 | *CHY* | *CHY* | *CHY* | *CHY* | *CHY* | *CHY* | *CHY* | *CHY* | *CHY* | *CHY* | *CHY* | *APT* | *CHY* |
