# Supplementary material for: Evaluation of New Reference Genes in Papaya for Accurate Transcript Normalization under Different Experimental Conditions
Source: PLoS One. 2012 Aug 31;7(8):e44405. doi: 10.1371/journal.pone.0044405 (PMC3432124; doi:10.1371/journal.pone.0044405)
Supplement: Table S4 — The results comparison of RefFinder and geNorm + Normfinder. (DOC) [file pone.0044405.s005.doc]

**Table S4.** The results comparison of RefFinder and geNorm + Normfinder

| 1-MCP treatment | | | | | | | Modified atmosphere packaging | | | | | |
| --- | --- | --- | --- | --- | --- | --- | --- | --- | --- | --- | --- | --- |
| **RefFinder** | | | | | | **Normfinder** | **RefFinder** | | | | | **Normfinder** |
| Rank | Delta CT | BestKeeper | Normfinder | Genorm | **Comprehensive ranking** | **+geNorm** | Delta CT | BestKeeper | Normfinder | Genorm | **Comprehensive ranking** | **+geNorm** |
| 1 | *TBP2* | *UBCE* | *TBP2* | *TBP2 | TBP1* | ***TBP2*** | ***TBP2*** | *EIF* | *RCA* | *EIF* | *EIF | TBP1* | ***EIF*** | ***EIF*** |
| 2 | *EIF* | *RAN* | *EIF* |  | ***TBP1*** | ***TBP1*** | *TBP1* | *RP* | *EF1* |  | ***TBP1*** | ***TBP1*** |
| 3 | *TBP1* | *SAMDC* | *TBP1* | *ACTIN* | ***ACTIN*** | ***ACTIN*** | *SAND* | *SAMDC* | *TBP1* | *SAND* | ***SAND*** | ***SAND*** |
| 4 | *ACTIN* | *ACTIN* | *ACTIN* | *PP2A* | ***EIF*** | ***EIF*** | *PP2A* | *EF2* | *SAND* | *CYP* | ***EF1*** | ***EF1*** |
| 5 | *PP2A* | *EF1* | *CYP* | *SAND* | ***UBCE*** | ***PP2A*** | *CYP* | *18SrRNA* | *SAMDC* | *PP2A* | ***PP2A*** | ***CYP*** |
| 6 | *SAND* | *TBP1* | *UBCE* | *EIF* | ***SAND*** | ***CYP*** | *EF1* | *EF1* | *PP2A* | *RPS* | ***CYP*** | ***PP2A*** |
| 7 | *CYP* | *SAND* | *EF1* | *CYP* | ***PP2A*** | ***SAND*** | *UBCE* | *RAN* | *RAN* | *UBCE* | ***SAMDC*** | ***UBCE*** |
| 8 | *UBCE* | *TBP2* | *SAND* | *EF1* | ***CYP*** | ***UBCE*** | *RPS* | *EIF* | *CYP* | *TBP2* | ***RCA*** | ***RPS*** |
| 9 | *EF1* | *EIF* | *PP2A* | *UBCE* | ***EF1*** | ***EF1*** | *TBP2* | *ACTIN* | *ACTIN* | *EF1* | ***RAN*** | ***TBP2*** |
| 10 | *SAMDC* | *CYP* | *RPS* | *RPS* | ***SAMDC*** | ***RPS*** | *APT* | *TBP1* | *TBP2* | *TUA* | ***UBCE*** | ***SAMDC*** |
| 11 | *RPS* | *18SrRNA* | *SAMDC* | *SAMDC* | ***RAN*** | ***SAMDC*** | *RAN* | *SAND* | *UBCE* | *APT* | ***TBP2*** | ***RAN*** |
| 12 | *RAN* | *PP2A* | *TUA* | *RAN* | ***RPS*** | ***RAN*** | *ACTIN* | *PP2A* | *APT* | *UBQ* | ***RPS*** | ***ACTIN*** |
| 13 | *TUA* | *RPS* | *RAN* | *TUA* | ***TUA*** | ***TUA*** | *TUA* | *CYP* | *18SrRNA* | *ACTIN* | ***ACTIN*** | ***APT*** |
| 14 | *RCA* | *APT* | *RCA* | *RCA* | ***RCA*** | ***RCA*** | *SAMDC* | *TBP2* | *RPS* | *RAN* | ***RP*** | ***TUA*** |
| 15 | *GAPDH* | *RCA* | *GAPDH* | *GAPDH* | ***18SrRNA*** | ***GAPDH*** | *UBQ* | *UBCE* | *EF2* | *18SrRNA* | ***18SrRNA*** | ***18SrRNA*** |
| 16 | *UBQ* | *TUA* | *EF2* | *UBQ* | ***GAPDH*** | ***UBQ*** | *18SrRNA* | *RPS* | *TUA* | *SAMDC* | ***EF2*** | ***UBQ*** |
| 17 | *18SrRNA* | *EF2* | *UBQ* | *18SrRNA* | ***UBQ*** | ***EF2*** | *EF2* | *APT* | *UBQ* | *EF2* | ***APT*** | ***EF2*** |
| 18 | *APT* | *UBQ* | *APT* | *APT* | ***APT*** | ***18SrRNA*** | *RCA* | *TUA* | *RCA* | *GAPDH* | ***TUA*** | ***RCA*** |
| 19 | *EF2* | *RP* | *18SrRNA* | *EF2* | ***EF2*** | ***APT*** | *RP* | *UBQ* | *RP* | *RCA* | ***UBQ*** | ***GAPDH*** |
| 20 | *RP* | *GAPDH* | *RP* | *RP* | ***RP*** | ***RP*** | *GAPDH* | *GAPDH* | *GAPDH* | *RP* | ***GAPDH*** | ***RP*** |
| 21 | *CHY* | *CHY* | *CHY* | *CHY* | ***CHY*** | ***CHY*** | *CHY* | *CHY* | *CHY* | *CHY* | ***CHY*** | ***CHY*** |
